# Estrogenicity of chemical mixtures revealed by a panel of bioassays

**DOI:** 10.1016/j.scitotenv.2021.147284

**Published:** 2021-09-01

**Authors:** Livia Gómez, Magdalena Niegowska, Anna Navarro, Luca Amendola, Augustine Arukwe, Selim Ait-Aissa, Stefania Balzamo, Salvatore Barreca, Shimshon Belkin, Michal Bittner, Ludek Blaha, Sebastian Buchinger, Maddalena Busetto, Mario Carere, Luisa Colzani, Pierluisa Dellavedova, Nancy Denslow, Beate I. Escher, Christer Hogstrand, Essa Ahsan Khan, Maria König, Kevin J. Kroll, Ines Lacchetti, Emmanuelle Maillot-Marechal, Liat Moscovici, Monica Potalivo, Isabella Sanseverino, Ricardo Santos, Andrea Schifferli, Rita Schlichting, Susanna Sforzini, Eszter Simon, Etai Shpigel, Stephen Sturzenbaum, Etienne Vermeirssen, Aldo Viarengo, Inge Werner, Teresa Lettieri

**Affiliations:** a European Commission, Joint Research Centre (JRC), Via E. Fermi 2749, 21027 Ispra, VA, Italy; b ARPA Lazio, Regional Agency for Environmental Protection, Via G. Saredo 52, 00173 Rome, Italy; c Department of Biology, Norwegian University of Science and Technology (NTNU), NO-7491 Trondheim, Norway; d French National Institute for Industrial Environment and Risks (INERIS), UMR-I 02 SEBIO, 60550 Verneuil-en-Halatte, France; e ISPRA - Environmental Metrology Unit, Via di Castel Romano 100, 00128 Rome, Italy; f ARPA Lombardia, Regional Agency for Environmental Protection, Via Rosellini 17, 20124 Milan, Italy; g Department of Plant and Environmental Sciences, The Alexander Silberman Institute of Life Sciences, The Hebrew University of Jerusalem, Jerusalem 91904, Israel; h RECETOX, Faculty of Science, Masaryk University, Kamenice 5, CZ62500 Brno, Czech Republic; i Federal Institute of Hydrology, Am Mainzer Tor 1, D-56068 Koblenz, Germany; j ISS-National Health Institute, Viale Regina Elena 299, 00161 Rome, Italy; k Center for Environmental & Human Toxicology, Department of Physiological Sciences, College of Veterinary Medicine, University of Florida, Gainesville, FL, USA; l Department Cell Toxicology, Helmholtz Centre for Environmental Research - UFZ, Permoserstraße 15, 04318 Leipzig, Germany; m Metal Metabolism Group, Department of Nutritional Sciences, Faculty of Life Sciences and Medicine, King's College London, 150 Stamford St, London SE1 9NH, UK; n Laboratório de Análises, Instituto Superior Tecnico, Universidade de Lisboa, Av. Rovisco Pais, 1049-001 Lisboa, Portugal; o Swiss Centre for Applied Ecotoxicology, Überlandstrasse 133, 8600 Dübendorf, Switzerland; p Institute for the Study of Anthropic Impacts and Sustainability in Marine Environment, National Research Council (CNR-IAS), Via de Marini 6, Genova 16149, Italy; q School of Population Health & Environmental Sciences, Faculty of Life Sciences & Medicine, King's College London, 150 Stamford Street, London, UK

**Keywords:** Bioassay, Chemical mixture, Environmental quality standard (EQS), Endocrine disrupting compound (EDC), Estrogenicity, Hormone mixture

## Abstract

Estrogenic compounds are widely released to surface waters and may cause adverse effects to sensitive aquatic species. Three hormones, estrone, 17β-estradiol and 17α-ethinylestradiol, are of particular concern as they are bioactive at very low concentrations. Current analytical methods are not all sensitive enough for monitoring these substances in water and do not cover mixture effects. Bioassays could complement chemical analysis since they detect the overall effect of complex mixtures. Here, four chemical mixtures and two hormone mixtures were prepared and tested as reference materials together with two environmental water samples by eight laboratories employing nine *in vitro* and *in vivo* bioassays covering different steps involved in the estrogenic response.

The reference materials included priority substances under the European Water Framework Directive, hormones and other emerging pollutants. Each substance in the mixture was present at its proposed safety limit concentration (EQS) in the European legislation.

The *in vitro* bioassays detected the estrogenic effect of chemical mixtures even when 17β-estradiol was not present but differences in responsiveness were observed. LiBERA was the most responsive, followed by LYES. The additive effect of the hormones was captured by ERα-CALUX, MELN, LYES and LiBERA. Particularly, all *in vitro* bioassays detected the estrogenic effects in environmental water samples (EEQ values in the range of 0.75–304 × EQS), although the concentrations of hormones were below the limit of quantification in analytical measurements. The present study confirms the applicability of reference materials for estrogenic effects' detection through bioassays and indicates possible methodological drawbacks of some of them that may lead to false negative/positive outcomes. The observed difference in responsiveness among bioassays – based on mixture composition - is probably due to biological differences between them, suggesting that panels of bioassays with different characteristics should be applied according to specific environmental pollution conditions.

## Highlights

• Estrogens are active at concentrations undetectable by chemical analysis.
• Bioassays detected estrogenic effects by other EDC than only hormones in water.
• Estrogenicity bioassays have different responsiveness to specific compounds.
• Some bioassays detected the additive effects of estrogens.
• Reference materials enlightened the ability of bioassays to detect combined effects.

## Graphical abstract

**Figure:**
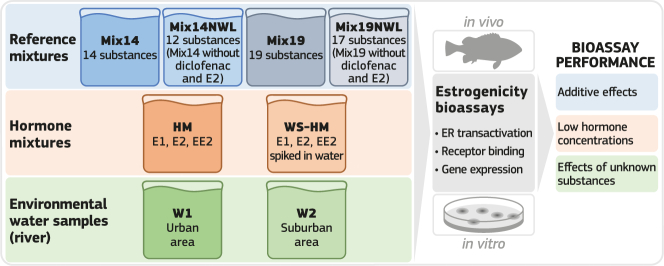

## 1 Introduction

Thousands of chemicals encompassing different classes of substances such as pesticides, pharmaceuticals, personal care and industrial products are discharged into the water environment from agricultural areas, urban settlements and industrial sites. These complex chemical mixtures spread in surface waters enable dynamic interactions between molecules and elicit combined effects that may be harmful to aquatic ecosystems and human health even when single substances occur at very low concentrations (ng and pg/L range). However, a strategy for water protection established by the European Union (EU) Directive 2000/60/EC (Water Framework Directive, WFD (EU, 2000)) only defines safety limit concentrations (Environmental Quality Standards, EQS) for a limited number of substances (Priority Substances; PS) of EU wide concern. For technical and economic reasons, it is not possible to analyze all the substances that occur in the aquatic environment. Water quality assessment is focused on chemical analysis of individual PS and river basin specific pollutants (RBSP), neglecting the real scenario of co-occurring pollutants and leaving the question of quantifying mixture effects unresolved. Effect-based methods (EBM), or bioassays, have been recommended (Brack et al., 2019; Escher et al., 2014; Kase et al., 2018; Könemann et al., 2018; Wernersson et al., 2015) to be applied together with chemical analysis for water quality monitoring in a regulatory context as they can cover mixture effects of co-occurring pollutants. To urgently address this issue, the European Commission's Joint Research Centre (JRC) performed a EU-wide exercise on chemical mixture effects (Carvalho et al., 2014), by exploring the use of artificial mixtures of known composition as reference materials in a panel of bioassays. The reference mixtures (RM), composed of 14 and 19 substances (Mix14 and Mix19, respectively) regulated by the European legislation (2008/105/EC, 2008; 2013/39/EU, 2013); and emerging pollutants (Carvalho et al., 2014) were tested in 35 *in vitro* and *in vivo* bioassays covering different endpoints and trophic levels. In selected tests, the mixtures exerted toxic action even when individual substances were present at levels considered safe (EQS level). The study provided a method for quantifying endpoint-based effects as EQS-fold concentrations. Additionally, differences in responsiveness in the detection of estrogenic activity in the mixtures were observed using four distinct *in vitro* bioassays (*i.e.*, YES, ERα-CALUX, MELN and human ERα competition assay) (Carvalho et al., 2014) pointing at inconsistency that may arise in risk assessment due to employed technique.

Estrogens are widely released to surface waters mainly due to medical therapy, contraceptive use and livestock-related agricultural practice (Adeel et al., 2017) and may cause adverse effects to sensitive aquatic species (*e.g.* fish). The three hormones, estrone (E1), 17β-estradiol (E2) and 17α-ethinylestradiol (EE2), are of particular environmental concern due to their constant discharge into surface waters *via* wastewater effluents and their bioactivity at very low (pg/L or ng/L) concentrations (Könemann et al., 2018). The above-mentioned hormones were included in the first Watch List (WL) (Carvalho et al., 2015; EU, 2015, EU, 2018) of the EU WFD (EU, 2015) which aims to gather high-quality Union-wide monitoring data on substances of concern for which existing monitoring data are not sufficient to perform the risk assessment. The proposed annual average Environmental Quality Standards (AA-EQS) for E1, E2 and EE2 are very low (400 pg/L, 400 pg/L and 35 pg/L, respectively) and current chemical analytical methods are not all sensitive enough for monitoring these substances in water, especially for EE2 and when matrix effect is also present (Kase et al., 2018; Loos, 2012). Bioassays are less affected by the sample matrix and can detect the estrogenic effects of water samples at ng or pg level, therefore they could be used as screening methods to complement chemical analysis (Könemann et al., 2018). Indeed, the use of *in vitro* and *in vivo* bioassays for monitoring estrogenic activity in water bodies has already been explored in very many studies (Brion et al., 2019; Carvalho et al., 2019; Jarošová et al., 2014b; Kase et al., 2018; Könemann et al., 2018; Kunz et al., 2017; Leusch et al., 2010; Mehinto et al., 2015; Wernersson et al., 2015). Bioassays are able to detect estrogenic compounds at lower concentrations than chemical analysis as shown by Könemann et al. (2018) and Kase et al. (2018) and peculiarly, they account for mixture effects. In these studies, the estrogenic activity of water samples quantified by the bioassays was expressed as 17β-estradiol (E2)-equivalence concentration (EEQ) (Escher et al., 2018b; Könemann et al., 2018; Kunz et al., 2017; Leusch et al., 2010; Simon et al., 2019) and compared to chemical analysis. The performance of the different bioassays was also explored (Brion et al., 2019; Könemann et al., 2018) and the obtained EEQ were comparable, even though differences were observed in responsiveness to specific estrogenic compounds (Könemann et al., 2018). Bioassays do not provide single substance-based measurements, but rather they provide a measure of the overall estrogenicity of water samples due to mixtures of known and unknown substances. E1, E2 and EE2 are the main contributors to estrogenic activity in surface water, however other substances such as alkylphenols, phthalates and bisphenol A (BPA) may also contribute to the overall estrogenic activity in the aquatic environment (Gonsioroski et al., 2020; Kunz et al., 2017).

Alongside these studies, the JRC launched a second EU-wide study, but this time to investigate endocrine-disrupting effects elicited by the most potent estrogenic compounds at proposed regulatory concentrations (EQS) in an expanded panel of bioassays and to assess environmental water samples. To this end, a reference hormone mixture (HM) including the WL hormones, E1, E2 and EE2, was prepared. In the absence of hormones, we also assessed estrogenic effects of other classes of substances with estrogenic activity confirmed by the European Chemicals Agency (ECHA), such as BPA and 4-nonylphenol (NP). Additionally, triclosan was included as a suspected endocrine-disrupting compound (EDC) (Dann and Hontela, 2011; von der Ohe et al., 2012). The main goal of the study following the first exercise (Carvalho et al., 2014) was to address the estrogenicity effects in a mixture, particularly looking at additive effects and the detection ability of the bioassays for substances at safety limit concentrations. Furthermore, this study provided a non–exhaustive snapshot of available *in vitro*/*in vivo* bioassays for the detection of endocrine disrupting effects. For the above purposes, nine *in vitro* and *in vivo* bioassays were employed to measure estrogenic effects as a response of binding to specific hormone receptors in transgenic yeast and human cell lines as well as the expression level of estrogen-related genes in fish and fish cell lines.

## 2 Material and methods

### 2.1 Reference materials and water samples

#### 2.1.1 Preparation of reference materials

Four reference mixtures (RM) (mixture 14, Mix14; mixture 19, Mix19, mixture 14 without WL substances, Mix14 NWL and mixture 19 without WL substances, Mix19 NWL) and two Hormone Mixtures (HM, water sample HM, WS-HM) were prepared and tested in the current study at a concentration equivalent to AA-EQS, designated EQS for simplification. The RM (Table 1a) were composed of PS, WL substances (Carvalho et al., 2015) and other emerging pollutants as described by Carvalho et al. (2014). Two additional RM (Mix14NWL and Mix19NWL) were prepared to test the capability of the panel of bioassays to detect estrogenic compounds when hormones are not present. The composition of these two RM was identical to Mix14 and Mix19 except they did not include the WL substances diclofenac and E2.

**Table 1:** Chemical composition of the reference mixtures Mix14, Mix19, Mix14 NWL, Mix19 NWL (a) and HM (b) tested in the current EU-wide estrogenicity study. ER, Estrogen Receptor, PS, Priority Substance; WL, Watch List.

a)									
Substance	CAS^a^	Regulation	Use	MoA and reported effects	AA-EQS^b^ μg/L	Mix14 1 × EQS μg/L	Mix19 1 × EQS μg/L	Mix14 NWL 1 × EQS μg/L	Mix19 NWL 1 × EQS μg/L
Atrazine	1912-24-9	PS	Herbicide	Photosystem II inhibitor	0.6^c^	0.6	0.6	0.6	0.6
Benzo[*a*]pyrene (BaP)	50-32-8	PS	By-product of incomplete combustion of organic material	Intercalation in DNA causing mutagenesis, carcinogenesis	0.00017^c^	0.00017	0.00017	0.00017	0.00017
Cadmium (Cd)	7440-43-9	PS	Industrial by-product: used in metal plating and to make pigments, batteries, and plastics. Insecticide	Indirect formation of reactive oxygen species depletion of glutathione, lipid peroxidation	0.08^c^	0.08	0.08	0.08	0.08
Chlorfenvinphos	470-90-6	PS	Insecticide	Inhibition of cholinesterase activity	0.1^c^	0.1	0.1	0.1	0.1
Chlorpyrifos	2921-88-2	PS	Insecticide	Inhibition of cholinesterase activity	0.03^c^	0.03	0.03	0.03	0.03
Bis(2-ethylhexyl) phthalate (DEHP)	117-81-7	PS	Plasticizer	DNA damage, carcinogenicity	1.3^d^	1.3	1.3	1.3	1.3
Diclofenac^f^	15307-79-6	1st WL	Pharmaceutical pain killer: non-steroidal anti-inflammatory drug (NSAID)	Can cause adverse hepatic effects in certain organisms	0.1^d^	0.1	0.1	–	–
Diuron	330-54-1	PS	Herbicide	Photosystem II inhibitor	0.2^c^	0.2	0.2	0.2	0.2
17ß-estradiol (E2)^f^	50-28-2	1st and 2nd WL	Natural estrogen	Natural estrogen	0.0004^d^	0.0004	0.0004	–	–
Fluoranthene	206-44-0	PS	Product of incomplete combustion	Causes mutagenesis, carcinogenesis	0.0063^c^	0.0063	0.0063	0.0063	0.0063
Isoproturon	34123-59-6	PS	Herbicide	Photosystem II inhibitor	0.3^c^	0.3	0.3	0.3	0.3
Nickel (Ni)	7440-02-0	PS	Industry, preparation of alloys	Depletion of glutathione levels, binds to sulfhydryl groups of proteins, carcinogenicity	4^c^	4	4	4	4
4-nonylphenol (NP)	25154-52-3	PS	Mostly used for the production of surfactants (nonylphenol ethoxylates)	Endocrine disruptor	0.3^c^	0.3	0.3	0.3	0.3
Simazine	122-34-9	PS	Herbicide	Photosystem II inhibitor	1^c^	1	1	1	1
Carbamazepine	298-46-4	Other emerging pollutants	Pharmaceutical (anti-epileptic, mood-stabilizing drug)	Teratogenicity	0.5^e^	–	0.5	–	0.5
Sulfamethoxazole	723-46-4	3rd WL	Pharmaceutical (antibiotic)	Interferes with folic acid synthesis	0.6^e^	–	0.6	–	0.6
Triclosan (Irgasan)	3380-34-5	Other emerging pollutants	Anti-bacterial and antifungal agent used in cosmetics and detergents	Inhibition of cellular efflux pumps	0.02^e^	–	0.02	–	0.02
*N*,*N*-diethyl-*meta*-toluamide (DEET)	134-62-3	Other emerging pollutants	Insect repellent	Affects insect odorant receptors, inhibits cholinesterase activity (nervous system)	41^e^	–	41	–	41
Bisphenol A (BPA)	80-05-7	Other emerging pollutants	Plasticizer	ER agonist	1.5^e^	–	1.5	–	1.5


b)						
Substance	CAS^a^	Regulation	Use	MoA and reported effects	AA-EQS^b^ μg/L	HM 1 × EQS μg/L
Estrone (E1)	53-16-7	1st and 2nd WL	Natural estrogen	Natural estrogen	0.0004^g^	0.0004
17ß-estradiol (E2)	50-28-2	1st and 2nd WL	Natural estrogen	Natural estrogen	0.0004^c^	0.0004
17α-ethinylestradiol (EE2)	57-63-6	1st and 2nd WL	Synthetic estrogen	Synthetic estrogen	0.000035^d^	0.000035

a Chemical Abstracts Service.

b Annual Average EQS.

c According to European Directive 2013/39/EU (EU, 2013).

d From COM 2011-876 (COM, 2011).

e Proposal from Ecotox Centre, Switzerland.

f Substances not included in Mix14 NWL and Mix19 NWL.

g From EU. CID 2015/495 and EU. CID 2018/840 (EU, 2015, EU, 2018).

To test the ability of the bioassays to detect additive effects of estrogenic hormones, a HM was prepared. The HM contained the three WL hormones (Table 1b).

For each mixture, a 10,000-fold concentrated reference material was prepared, with organic compounds in methanol and inorganic chemicals (metals) in 2% nitric acid according to ISO 17034 (ISO 17034, 2016). The HM was prepared at two different concentrations, 10,000-fold and 1000-fold concentrated. The 10,000-fold concentrated HM was used as reference material while the 1000-fold concentrated HM was spiked in 1 L ultrapure water (resistivity of >18.2 MegaOhm/cm^2^) to simulate an environmental water sample (WS-HM) at 1 × EQS concentration. The sample was then enriched by solid phase extraction (SPE). Each laboratory used its routine protocol and methodology including SPE blank extraction controls when required (see Section 2.2).

The chemicals used for the preparation of the reference materials were of ≥98% purity, whereas for Benzo(*a*)pyrene (BaP) and *N,N*-diethyl-meta-toluamide (DEET) the purity was ≥96 and ≥97%, respectively. Both stability and homogeneity were assessed as recommended by the ISO Guide 35 (ISO Guide 35, 2017). The short-term stability was assessed according to an isochronous study in order to simulate problematic transport or storage conditions with a reference temperature of −20 °C and 4 °C and a test temperature of 24 °C for up to 12 weeks. This approach minimizes the variations in analytical response as a function of time. All the substances were determined by means of UHPLC/MS-MS by the Italian Institute for Environmental Protection and Research (ISPRA) except E1, E2 and EE2 that were determined by the Regional Environmental Protection Agency of Lombardy (ARPA Lombardia, Italy). All the analytes were stable and homogenous in all the reference materials. A dedicated section including all details on the preparation of the reference materials, and the stability and homogeneity studies is included in the Supplementary Information (Supplementary materials and methods).

#### 2.1.2 Water sample collection and analysis

**Fig. 1:**
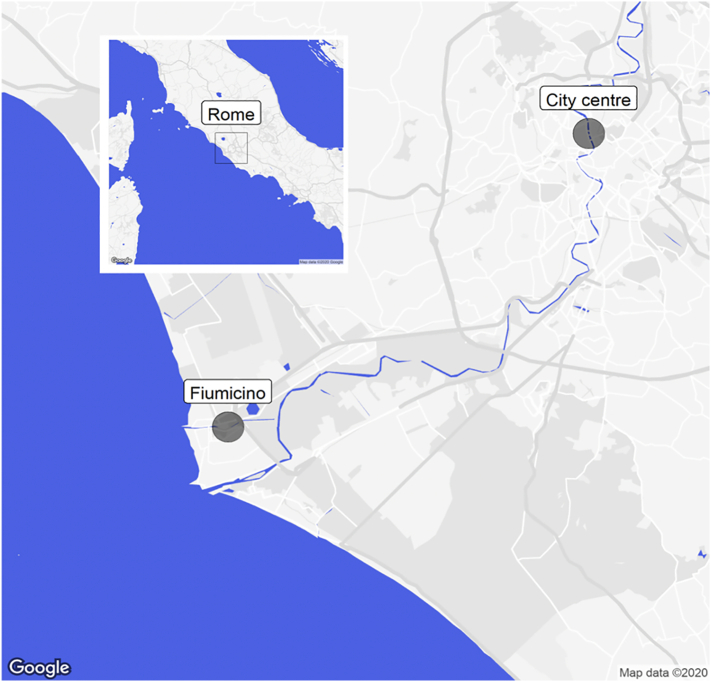
Location of the sampling sites. Sampling site 1: city centre, ARPA-Lazio station located in *Via* di Ripetta, Rome (coordinates: 41.912351, 12.471794; date: 24/10/2018; time: 02.02 p.m.; temperature: 17 °C; pH: 7.62; conductivity: 1223.5 μS/cm; oxygen saturation: 89.4%). Sampling site 2: Fiumicino, located in *Via* Ugo Baistrocchi, Fiumicino-Rome (coordinates: 41.770877, 12.24790; date: 24/10/2018; time: 11.20 a.m.; temperature: 16.4 °C; pH: 7.51; conductivity: 4907 μS/cm; oxygen saturation: 55%).

Two sampling sites were selected along the Tiber river (Rome, Italy) for the water sample (WS) collection (Fig. 1). The first sampling site is located in the city center (sampling site 1; water sample 1, WS1). The second sampling site is located 30 km downstream from sampling site 1 in Fiumicino, close to the river mouth (sampling site 2; water sample 2, WS2). The WS were collected in October 2018 from surface water (20 cm water depth) in 1 L capacity wide-mouth Boston round high-density polyethylene (HDPE) bottles. After collection, the samples were frozen (−80 °C) and distributed to the laboratories. The main physicochemical parameters such as temperature, pH, conductivity (μS/cm), and dissolved oxygen (mg/L) (or oxygen saturation in %) were measured during sampling (on-site) using a multi-parameter meter (Orion Star A329, ThermoFisher Scientific, USA). WS were tested in the bioassays after SPE at different concentrations expressed as relative enrichment factor (REF), accounting for enrichment during the extraction and possible dilution during the bioassay analyses, following the laboratory's routine procedure.

The individual components of the RM (Mix14, Mix19) were analyzed in the WS (Table 1a) using pertinent internal standards. Detailed information on the analytical methods can be found in the supplementary information (Supplementary materials and methods). Briefly, BaP, chlorpyrifos, DEHP, fluoranthene, NP, triclosan and BPA were determined by GC–MS/MS after liquid-liquid microextraction (1 L of sample with 4 mL of toluene), evaporation to dryness and addition of toluene as internal standard to 100 μL of final volume.

Atrazine, chlorfenvinphos, diclofenac, diuron, E2, isoproturon, simazine, carbamazepine, sulfamethoxazole and DEET were determined by LC-MS/MS electrospray ionization (ESI, neg/pos) direct analysis of water after addition of methanol (5 mL in 1 L sample), filtration on 0.45 μm membrane, addition of atrazine-D5 as an internal standard for ESI positive mode and MCPA-D3 for ESI negative mode, and carbamazepine-C13 for carbamazepine determination. The extraction (only for E2) was carried out from the extraction of the 1 L sample with SPE-DEX (Horizon Technology, Italy) using a sandwich of HLB + C18 disks and elution with 20 mL of ethyl acetate-methanol-MTBE (1:1:1) mix. The solution was then evaporated to 500 μL and analyzed in ESI negative mode. Finally, the metals Ni and Cd were analyzed by ICP-MS following the EPA 6020B method.

### 2.2 Bioassays

Nine bioassays covering estrogenicity were used to test the reference materials. The list of *in vitro* and *in vivo* bioassays used by the partner laboratories in the current EU-wide estrogenicity study is provided in the supplementary information (Table S1).

#### 2.2.1 *In vitro* human and yeast cell-based estrogenicity bioassays

The *in vitro* bioassays based on cells (human or yeast) expressing the human estrogen receptor (hER) used in this work have been grouped as cell-based *in vitro* estrogenicity bioassays.

##### 2.2.1.1 Lyticase yeast estrogenic screen

The Lyticase-based Yeast Estrogen Screen (LYES) was performed according to (ISO 19040-1, 2018) with minor modifications. Yeast cells were pre-cultured in growth medium for 22 ± 1 h (30 °C) before the beginning of the test procedure. The assay was conducted on 96-well microtiter plates. E2 in ethanol served as a positive control, while ethanol (80 μL/well, *n* = 8 wells/plate) and the assay medium with the yeast cells not exposed to any RM, WS extract or organic solvent were included as negative controls. Positive control and samples were assessed in a 1:2 dilution series over eight wells. The highest concentration of E2 was 142 ng/L. On the test day, dilution series of the positive control, mixtures and WS extracts were prepared in glass vials, after complete ablation of the solvent, resuspended in 80 μL of sterile nanopure water. Finally, 40 μL of yeast cell suspension was added to each well. After 18 ± 1 h incubation (30 °C), cell density was measured at 600 nm with Synergy 4 microtiter plate photometer (BioTek, USA) for the determination of growth inhibition and/or cytotoxic effects. For the measurement, 30 μL of cell suspension of each well were transferred to a new plate, where 50 μL of lysis mix containing chlorophenol red-β-D-galactopyranoside (CPRG) was added. After 60 min of incubation (30 ± 1 °C under constant agitation), the color reaction was measured at 540 nm (Schultis and Metzger, 2004). The highest E2 concentration tested was 142 ng/L. The RM and HM were tested at concentrations ranging from 833 to 3.3 × EQS, while the WS extracts were tested at concentrations ranging from 290 to 0.5 REF. SPE was performed according to the Ecotox Centre standard operating procedure (SOP) and SPE blanks were also tested in the bioassay (Simon et al., 2019). All extracts and reference compounds were analyzed in duplicate or triplicate.

##### 2.2.1.2 ERα-CALUX®

For the ERα-CALUX testing, the solvent of the RM, HM and WS extracts was completely evaporated under a gentle nitrogen stream. The extracts were then redissolved in 25 μL of dimethyl sulfoxide (DMSO) and dilution series were prepared in 1.2 mL amber vials with integrated micro-insert (optimally: 1-3-10-30-60-100-300-600-1000-3000×).

The assay was performed based on the ISO standard (ISO 19040-3, 2018; Sonneveld et al., 2005). Briefly, the U2OS cells (human bone osteosarcoma) were seeded into 96-well plates with growth medium (without phenol red, supplemented with dextran-coated charcoal stripped serum). After 24 h of incubation (37 °C, 5% CO_2_), the growth medium was replaced by the exposure medium containing the RM, HM and WS extracts (at 0.1% DMSO) for agonistic activity testing. After 24 h of incubation, the exposure medium was removed and the cells were lysed in 30 μL of Triton lysis buffer. The amount of luciferase activity was quantified using a luminometer (MicroLumat Plus, Berthold Technologies, Switzerland). On all plates, a dose-response curve of the reference compound was included for the quantification of the response to E2. The highest E2 concentration tested was 27 ng/L. The RM and HM were assessed at concentrations ranging from 200 to 0.02 × EQS, while the WS extracts were tested at concentrations ranging from 8.7 to 0.003 REF. All RM, HM, WS extracts and reference compound were analyzed in triplicate. SPE was performed according to the Ecotox Centre SOP and SPE blanks were also tested in the bioassay (Simon et al., 2019).

##### 2.2.1.3 hERα-HeLa-9903 cells

The (anti-)estrogenicity of RM, HM and WS extracts was assessed on stably transfected human cell line hERα-HeLa-9903. The assay was performed according to the modified guideline OECD 455 (OECD, 2015). Briefly, the cells were cultured in Dulbecco's Modified Eagle's Medium (DMEM) (Sigma-Aldrich, Czechia) enriched by 10% Fetal Bovine Serum (FBS) (Biochrom GmbH, Germany) and maintained at humidified incubator (37 °C, 5% CO_2_). After 24 h, cells were seeded at a density of 2 × 10^4^ cells per well on a 96-well plate (Greiner Bio-One GmbH, Austria) in DMEM supplemented with 10% charcoal-stripped FBS (Sigma-Aldrich, Czechia). Cells were exposed to dilution series of RM, HM, WS extracts, E2 calibration standards (dilution series 1 to 136 ng/L) and solvent control in three replicates. For anti-estrogenity tests, co-exposure of samples with a 9 ng/L E2 sample was performed. The final concentration of the solvent (methanol) was 0.5% *v*/v. After 24 h exposure, the intensity of luminescence was measured on Luminoskan Ascent luminometer (ThermoFisher Scientific, USA) using Steady-Glo® Kit (Promega, USA).

Samples were diluted with 200 × culture medium to obtain the highest concentration of solvent (0.5% v/v). RM samples were diluted and organic and inorganic samples were mixed in the culture medium. Subsequently, 100 μL of the obtained mixtures were added to cell cultures. The RM and HM were tested at concentrations ranging from 1.56 × EQS to 50 × EQS. Solvent and negative controls were also included. After SPE, WS were tested at concentrations ranging from 10 REF to 0.313 REF. A blank control for the SPE was tested in the hERα-HeLa-9903 cells alongside with the WS. The sample was prepared by extracting ultra pure water (resistivity of >18.2 MegaOhm/cm^2^) prepared by the device Purelab Flex 3 and processed in the same way as the WS.

##### 2.2.1.4 MELN cells

The ER-mediated activities of the RM, HM and WS extracts were monitored by using the MELN reporter cell line. The MELN cell line was obtained by stable transfection of MCF-7 human breast cancer cells using an ERE-βGlob-Luc-SVNeo plasmid (Balaguer et al., 2001). The cell line was routinely cultured in DMEM containing phenol red, supplemented with 5% FBS at 37 °C under 5% CO_2_ humidified atmosphere.

Cells were seeded into 96-well plates at a density of 5 × 10^4^ cells/well in phenol red-free DMEM supplemented with 3% stripped serum. Twenty-four hours after plating, cells were dosed with various dilutions of mixtures, solvent control and positive control. WS were concentrated by SPE using OASIS-HLB cartridges eluted with dichloromethane-MeOH (v:v) and then the extracts transferred into DMSO. A blank control using milli-Q in parallel to the samples. The blanks were negative in MELN cells. A dose-response curve of the reference compound (E2) was also prepared. Upon overnight exposure (18 h), 0.3 mM of D-luciferin was added to the wells. After 5 min, the luminescence was measured in living cells for 2 s per well using luminometer (Synergy H4, Biotek). The RM and HM were tested at concentrations ranging from 0.01 × EQS to 50 × EQS. After SPE, the WS extracts were tested at concentrations ranging from 0.00252 REF to 13.5 REF. Effect data were determined as a percentage of maximal luciferase activity induced by 2.7 μg/L E2, after subtraction of the background signal in untreated cells.

##### 2.2.1.5 ERα GeneBLAzer

GeneBLAzer™ ERα-UAS-*bla* GripTite™ cells (ThermoFisher Scientific, USA) contain the ligand-binding domain (LBD) of hERα fused to the DNA-binding domain of GAL4 stably integrated in the GeneBLAzer®UAS-bla GripTite™ cell line expressing β-lactamase reporter gene under the transcriptional control of an upstream activator sequence (UAS). When an agonist binds to the LBD of the GAL4 (DBD)-ERα (LBD) fusion protein, the protein binds to the UAS, resulting in expression of β-lactamase.

The experimental method is detailed in König et al. (2017) with the cytotoxicity quantification described in Escher et al. (2019) (more details in the SI). Briefly, the cells were seeded in 384-well plates and incubated between 4 and 24 h at 37 °C and 5% CO_2_. Dilution series of the reference compound, RM, HM and WS extracts were added to the cells and incubated for 48 h at 37 °C and 5% CO_2_. The cellular response was measured by fluorescence. The highest E2 concentration tested was 4 μg/L. The RM and HM were tested at concentrations ranging from 500 to 0.07 × EQS, while the WS extracts were tested at concentrations ranging from 312 to 0.01 REF. Every assay was performed at least twice to take into account the day-to-day variability of the bioassays.

##### 2.2.1.6 Planar yeast estrogenic screen

The Yeast Estrogen Screen (YES) based on yeast cells according to McDonnell et al. (1991), was performed on silica-surface plates (pYES) for high performance thin-layer chromatography (HPTLC) after chromatographic separation of the sample (Buchinger et al., 2013). The yeast cells were applied by spraying (Schoenborn et al., 2017). After a 3 h exposure of the cells at 30 °C, the induction of the reporter system based on *lacZ* encoding the enzyme β-galactosidase was detected by the cleavage of the artificial substrate 4-methylumbelliferyl β-D-galactopyranoside (MUG) resulting in a fluorescent signal.

An HPTLC plate was applied with different volumes (*i.e.*, 2, 5, 10; 15; and 20 μL) of Mix19 at a concentration of 10,000 × EQS and a solution of the same mixture diluted 10 times in methanol (1000 × EQS). The water samples WS1 and WS2 were enriched 1000-fold by SPE using an Oasis HLB-cartridge (Waters Corporation, USA). Methanol was used for the elution of the SPE-cartridge. 25 μL of this SPE extract were applied to the same HPTLC plate that was used for the Mix19. A reference mix prepared by the laboratory containing 4-isononylphenol, E1, EE2, E2 and estriol (E3) was applied as a further reference. The HPTLC-plates were developed with two different mobile phase containing chloroform/ethyl acetate/petroleum fraction 55:20:25 (v/v/v) or ethylacetate/hexane 50:50 (v/v). After chromatography, the developed HPTLC plates were analyzed by pYES (Schoenborn et al., 2017).

Different volumes of the 10,000 × EQS HM standard and a 1:10 dilution in methanol were applied to an HPTLC plate (*i.e.*, 2, 5, 10, 15, and 20 μL) and analyzed by pYES subsequently. Based on the mean peak areas of the detected signals, dose-response curves were prepared and the data were fitted using a five-parametric sigmoidal function.

Dose-response relationships for the estrogenic compounds E1, E2 and EE2 were generated by using the 10,000 × EQS HM.

The 1000 × EQS HM was used to spike 1000 mL of Milli-Q water as described in Section 2.1.1. The sample was enriched 1000-fold by an SPE using an Oasis HLB-cartridge. Methanol was used for the elution of the SPE-cartridge. 25 μL of this SPE-extract were applied to an HPTLC plate together with various volumes of the 10,000 × EQS HM for calibration. 25 μL of a procedure blank (*i.e.*, unspiked Milli-Q water enriched in methanol 1000-fold by the same procedure) were applied as the negative control. Based on the detected signal intensity the compounds E1, E2 and EE2 were quantified using the individual dose-response relationships by inserting the signal intensity in the inverse fitting function.

#### 2.2.2 Ligand Binding Estrogen Receptor Assay

The ligand-binding estrogen receptor assay (LiBERA) was used to test the binding of the different mixtures to the human ERα. It is a modified version of the PolarScreen™ ERα green assay developed by Life Technologies that uses a wild-type ERα ligand-binding domain (wtERα^LBD^) instead of the full-length protein provided with the kit, according to Ferrero et al. (2014). This assay has been already used to test the effects of chemical mixtures by Carvalho et al. (2014) named wtERα^LBD^ binding assay. The assay is based on the displacement of the Fluoromone ES2 from the ER receptor by competitor molecules and a consequent decrease in the maximum fluorescence signal. The intensity of the fluorescence polarization (P) signal was measured with an Infinite 200 Pro multimode plate reader (Tecan, Switzerland). Dose-dependent responses ranging from 0.01 to 200 × EQS, 0.2 to 4000 × EQS, and 0.04–20 REF for the RM, HM and WS (respectively) were investigated. The data were fitted to a sigmoidal one-site competition four parameters logistic curve with GraphPad Prism version 8.4.3 (GraphPad Software, USA). The fit provided the IC_10_ and IC_50_ value (concentration of test compound, mixture or WS required to reduce the maximum polarization value to 90% and 50%).

#### 2.2.3 Gene expression analysis by quantitative real-time PCR

##### 2.2.3.1 *Poeciliopsis lucida* hepatocellular carcinoma derived PLHC-1 cell line exposure

PLHC-1 cell line was obtained from American Type Culture Collection (ATTC CRL 2406) (Ryan and Hightower, 1994). The cells were grown at 30 °C in phenol red-free DMEM supplemented with 2 nM l-glutamine, 1% penicillin-streptomycin and 5% FBS in a 5% CO_2_ humidified atmosphere. Initially, cells were grown in a 75-cm^2^ culture flask to get 75% monolayer confluence, then harvested with trypsin (0.05%) and EDTA (0.53 mM) in phosphate-buffered saline solution (PBS) and diluted 3–4 times.

For exposure, cells were seeded at the rate of 3 × 10^4^ cells per well in a 24-well plate and allowed to attach. After 24 h of incubation, growth medium was substituted with new medium supplemented with RM or HM, at different dilutions (1×, 2×, 5×, 10×, 20×, 40×, and 50×). Negative and solvent controls were run in parallel to quantify the changes in expression levels of estrogen receptor alpha (*erα*) and vitellogenin (*vtg*) genes. Each dilution of exposure mixtures was performed in six parallel replicates. Cells were exposed for 48 h in a 5% CO_2_ humidified incubator.

After exposure, total RNA was extracted using Direct-zol RNA MiniPrep isolation kit (Zymo Research, USA) according to instruction provided by the kit manufacturer, followed by RNA quantification using the NanoDrop® ND-1000 UV–vis Spectrophotometer (ThermoFisher Scientific, USA). For cDNA preparation, 0.2 μg of DNAse-treated total RNA was used as a template and poly-T primer from the iScript cDNA synthesis kit (Bio-Rad, USA). The procedure of reverse transcription was performed in a strict temperature profile of thermal cycler (Bio-Rad): 5 min at 25 °C, 30 min at 42 °C, and 5 min at 85 °C.

Specific primer pair sequences (Table S2) for *erα* and *vtg* genes were amplified using the Mx3000P real-time PCR machine (Stratagene, USA). The primer pairs were tested and shown to amplify a single PCR product of the expected size for individual genes. A parallel control, lacking cDNA template was used to validate the specificity and target sequence amplification. PCR program included an enzyme activation step at 95 °C (4 min) followed by 40 cycles of 95 °C (15 s), 60 °C (30 s) and 72 °C (15 s) with the last step temperature profile: 95 °C (60 s), 65 °C (30 s) and 95 °C (30 s). The expression of each gene was determined by following the well-validated procedure of absolute quantification in the partner laboratory (Mortensen and Arukwe, 2007). A known amount of plasmid cloned with an amplicon of interest was used to generate a standard curve. The pre-made standard plot of cycle threshold (Ct) *versus* log copy number was used to quantify the expression of the target gene in unknown samples.

##### 2.2.3.2 Fathead minnow early life stage exposure

Fathead minnow (*Pimephales promelas*) juveniles (10 days post hatch) were dispersed randomly into 10 mL beakers with 6 fish per vessel and quadruplicate beakers per condition. 5 mL of the test solution (RM or HM) was added to each beaker. The control water and the test solutions were renewed by 50% daily. The fish exposures were continued for 3 days. At the end of the 3-day period, juveniles were euthanized with MS222 and individual fish were placed into RNAlater (ThermoFisher Scientific, USA) for total RNA extraction.

Exposure mixtures were prepared in small volumes (2–4 mL) and in methanol. Exposure experiments were conducted in water adjusted to 20% Hank's salt solution (27 mM NaCl, 1 mM KCl, 0.05 mM Na_2_HPO_4_, 0.09 mM KH_2_PO_4_, 0.25 mM CaCl_2_, 0.2 mM MgSO_4_, 41 mM NaHCO_3_). Methanol concentration was adjusted to 1% for all doses including a vehicle control. The 250 × EQS samples required a solvent exchange from methanol to ethanol/methanol (60/40) since the former was at toxic levels for fish. Only glass containers and pipets (Hamilton syringes) were used in this study to prevent compound losses to plastics due to sorption.

For total RNA purification, an in-house optimized standard method was used. Briefly, fish from each beaker were individually ground with a hand homogenizer in RNA STAT-60™ (Tel-Test, Inc., USA) following the manufacturer's protocol. RNA was quantified using the NanoDropTM spectrometer (ThermoFisher Scientific, USA), re-dissolved in RNAsecure™ and stored at −20 °C. All individuals were processed separately. For qPCR, *rpL7* was used as the housekeeping gene control and *vtg* as a biomarker for estrogenic chemicals.

### 2.3 Data evaluation

Biological activities (*i.e.* receptor transactivation or receptor binding) of the RM, HM, and WS extracts were determined by the *in vitro* bioassays and expressed as biological equivalence concentrations (BEQ), representing the concentration of the positive control which elicits the same effect as the mixture or the WS (*e.g.* E2-equivalent concentration (EEQ)) (Escher et al., 2014; Kunz et al., 2017; Simon et al., 2019) according to the individual protocols of the test performers.

To derive BEQ, concentration-response relationships of reference compounds and a dilution series of the mixtures, WS extracts and blanks were fitted to a four parameter non-linear regression curve (GraphPad Prism version 8.4.3) as previously described for (LYES, ERα-CALUX, hERα-HeLa-9903 and MELN) (Escher et al., 2014; Escher et al., 2008; Kunz et al., 2017; Neale et al., 2017; Simon et al., 2019), to a linear concentration-response curve for ERα–GeneBlazer (Escher et al., 2018b), or to a sigmoidal one site competition four-parameters logistic curve (GraphPad Prism version 8.4.3) for LiBERA (Ferrero et al., 2014). The fit provided the EC_y_ (*e.g.* EC_10_, EC_50_ or IC_10_ and IC_50_) values, the concentrations causing y% response (*e.g.* 10% and 50% effect or 10% and 50% inhibition), respectively.

The BEQ of the sample or mixture were determined by applying the following formula (1), while EEQ were determined by applying formula (2):

(1) $${\mathrm{BEQ}}_{\text{sample}}=\frac{{\mathrm{EC}}_{y}\;\text{or}\;{\mathrm{IC}}_{y}\;\left(\text{reference substance or mixture}\right)}{{\mathrm{EC}}_{y}\;\text{or}\;{\mathrm{IC}}_{y}\;\left(\text{sample}\right)}$$

(2) $${\mathrm{EEQ}}_{\text{sample}}=\frac{{\mathrm{EC}}_{y}\;\text{or}\;{\mathrm{IC}}_{y}\;\left(E2\right)}{{\mathrm{EC}}_{y}\;\text{or}\;{\mathrm{IC}}_{y}\;\left(\text{sample}\right)}$$

The test concentrations of the mixtures were expressed as EQS multipliers (× EQS). For more convenient comparison the concentration of the reference compound E2 was expressed in the same units (× EQS). For this purpose, E2 concentrations were transformed to EQS multipliers considering that 0.4 ng E2/L is equivalent to 1 × EQS. The WS concentrations were expressed as REF that incorporates the enrichment by SPE and the dilution of the extract in the bioassay. Therefore, REF is a measure of how much a WS would have to be enriched (REF > 1) or diluted (REF < 1) to achieve a given effect and is determined as follows:

(3) $$\mathrm{REF}={\text{Dilution factor}}_{\text{bioassay}}{\text{/Enrichment factor}}_{\text{SPE}}$$

where

(4) $${\text{Enrichment factor}}_{\text{SPE}}={\text{V}}_{\text{water}}{\text{/V}}_{\text{extract}}$$

and

(5) $${\text{Dilution factor}}_{\text{Bioassay}}=\text{Volume of extract added to bioassay/Total volume of bioassay}$$

The estrogenic effect of the WS were expressed as EEQ (E2-equivalents, E2-Eq), and those of the mixtures also mixture-BEQ, *i.e.*, Mix14EQ (Mix14-equivalents, Mix14-Eq), Mix19EQ (Mix19-equivalents, Mix19-Eq) and HMEQ (HM-equivalents, HM-Eq).

The E2 concentration causing the 10% or 50% of effect was expressed in ng/L, and in EQS multipliers (× EQS) by dividing the concentration in ng/L by the E2 EQS (0.4 ng/L). To compare the performance of the different cell-based estrogenicity bioassays, the results were expressed as E2 equivalents (EEQ) in ng E2-Eq/L and × EQS E2-Eq. The estrogenic activity of the WS was also expressed as Mix14, Mix19 and HM equivalent concentration (Mix14EQ, Mix19EQ and HMEQ, respectively) following formula (1), by dividing the EC_10_ or EC_50_ of the mixtures by the EC_10_ or EC_50_ of the WS (*e.g.* Mix14EQ = EC_10Mix14_/EC_10WS_).

For each bioassay the predicted EEQ (considering concentration addition) of the WS-HM was also determined. The predicted EEQ values of the WS-HM were calculated as follows:

(6) $${\mathrm{EEQ}}_{\text{Pred}\left(\mathrm{WS}-\mathrm{HM}\right)}={\mathrm{REP}}_{E1}\left[E1\right]+{\mathrm{REP}}_{E2}\;\left[E2\right]+{\mathrm{REP}}_{\mathrm{EE}2}\;\left[\mathrm{EE}2\right]$$

where

REP is the relative estrogenic potency. For ERα-CALUX, ERα-GeneBLAzer, hERα-HeLa0093 and MELN the REF values were taken from Könemann et al. (2018) and for LYES they were calculated from Schultis and Metzger (2004).

Finally, we determined the WS concentration (× EQS) by plotting on the reference mixture concentration-response curve the respective % effect value obtained through the bioassays (supplementary information, Fig. S1). First, the % of effect corresponding to a concentration 1 REF (equivalent to a not diluted or concentrated sample) in each bioassay was determined from the concentration-response curves prepared with the environmental water samples (WS1 and WS2) and the artificial water sample (WS-HM). Then, these values were used to interpolate the corresponding effect-concentration in the concentration-response curves prepared with the reference materials. These results are shown in the supplementary information (Table S5, Fig. S3).

## 3 Results

### 3.1 Chemical analysis of the water samples

The environmental WS collected from the Tiber river (Fig. 1, Section 2.1.2) were analyzed for the substances present in the RM (Table 1, Section 2.1.1). Table 2 shows the results of the chemical analysis. All the substances were present at concentrations lower than their limit of quantification (LOQ) and far below their individual proposed EQS, except BaP, fluoranthene, Ni and DEET in WS1, and Ni and sulfamethoxazole in WS2 that could be quantified below the EQS and triclosan in WS1 which concentration was slightly above the EQS. Moreover, the concentration of the WL hormones (E1, E2 and E2) in Rome sampling sites were below the EQS (E1 < 0.4 ng/L, E2 < 0.4 ng/L, EE2 < 0.035 ng/L). The data were provided by the Regional Environmental Protection Agency of Lazio (ARPA Lazio, Italy).

**Table 2:** Water sample analysis and environmental quality standards (EQS) of the analyzed substances expressed as concentrations in μg/L. The two water samples (WS1, WS2) collected in fall and spring (October and April), with October data reported here. < indicates concentrations below the limit of quantification (LOQ).

Substance	October		μg/L
	WS1 (Rome) μg/L	WS2 (Fiumicino) μg/L	
Atrazine	<0.05	<0.05	0.6
Benzo[*a*]pyrene (BaP)	0.00070	<0.00005	0.00017
Cadmium (Cd)	<0.05	<0.05	0.08
Chlorfenvinphos	<0.05	<0.05	0.1
Chlorpyrifos	<0.009	<0.009	0.03
Bis(2-ethylhexyl) phthalate (DEHP)	<0.39	<0.39	1.3
Diclofenac	<0.05	<0.05	0.1
Diuron	<0.05	<0.05	0.2
17ß-estradiol (E2)	<0.0004	<0.0004	0.0004
Fluoranthene	0.0005	<0.0002	0.0063
Isoproturon	<0.05	<0.05	0.3
Nickel (Ni)	1.5	1.0	4
4-nonylphenol (NP)	<0.09	<0.09	0.3
Simazine	<0.05	<0.05	1
Carbamazepine	<0.1	<0.1	0.5
Sulfamethoxazole	<0.1	0.07	0.6
Triclosan	0.05	<0.02	0.02
*N*,*N*-diethyl-*meta*-toluamide (DEET)	0.6	<0.05	44
Bisphenol A (BPA)	<0.0015	<0.0015	1.5

### 3.2 Cell-based estrogenicity bioassays and ligand binding estrogen receptor assay

**Fig. 2:**
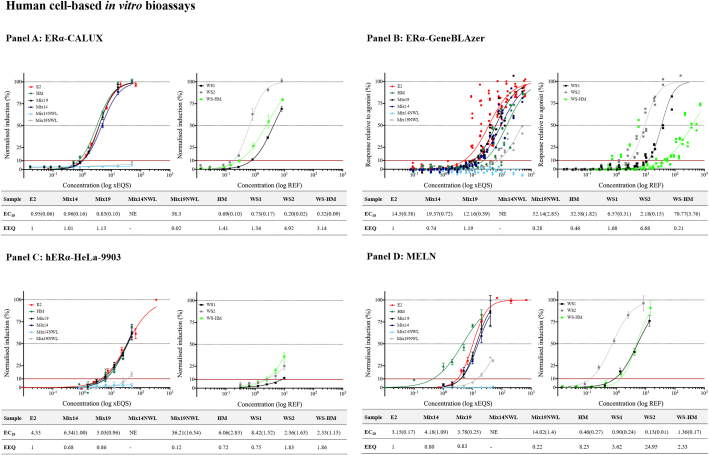
Results of testing the RM (Mix14, Mix19, Mix14 NWL, Mix19 NWL and HM), reference compound E2 and WS (WS1, WS2 and WS HM) on the human cell-based estrogenicity bioassays. Each panel shows the results of one bioassay, ERα-CALUX (panel A), ERα-GeneBLAzer (panel B), hERα-HeLa-9903 (panel C), and MELN (panel D). The left graph in each panel shows the dose-response curves obtained with the RM, HM and E2, while the right graph shows the dose-response curves of the WS. The concentrations are expressed as EQS-folds for the RM and E2, and REF for the WS. In each panel, the table below the graphs indicates EC_10_ values in × EQS or REF interpolated from the dose-response curves and the estrogenic potency compared to E2, expressed as EEQ (x EQS. E2-Eq). It was not possible to generate a complete dose-response curve for the WS1 in the case of hERα-HeLA9903 but the obtained values were fitted to a four-parameter non-linear regression curve from which the EC_10_ values were extrapolated. NE, no effect. The EC_10_ values are presented as mean with the standard deviation (SD) in brackets.

**Fig. 3:**
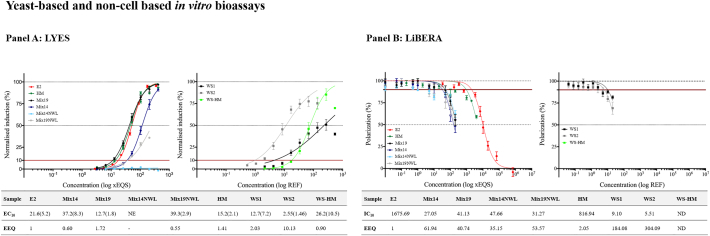
Results of testing the RM (Mix14, Mix19, Mix14 NWL, Mix19 NWL and HM), reference compound E2 and WS (WS1, WS2 and WS HM) on the yeast-based and the non-cell based estrogenicity bioassays. Each panel shows the results of one bioassay, LYES (panel A), LiBERA (panel B). The left graph in each panel shows the dose-response curves obtained with the RM, HM and E2, while the right graph shows the dose-response curves of the WS. The concentrations are expressed as EQS-folds for the RM and E2, and REF for the WS. In each panel, the table below the graphs indicates EC_10_ values for LYES and IC10 values for LiBERA in × EQS or REF interpolated from the dose-response curves, and the estrogenic potency compared to E2, expressed as EEQ (x EQS. E2-Eq). In the case of LiBERA, it was not possible to obtain a complete dose-response curve for the WS at the tested concentrations, the IC_10_ values were interpolated from the fitted sigmoidal one site competition four-parameter logistic curve. ND, not determined; NE, no effect. The EC_10_ values are presented as mean with the standard deviation (SD) in brackets.

The estrogenicity of the RM, HM and WS (see Section 2.1.1 and Table 1 for details on the mixture composition) was determined by six *in vitro* bioassays. The results are summarized in Fig. 2 and Fig. 3 where outcomes of each bioassay are shown in single panels, with the right graphs showing the dose-response curves obtained with the reference materials (RM, HM, E2) and the left graphs representing plots obtained for the WS (WS1, WS2 and WS-HM). Fig. 2 shows the results obtained when testing the mixtures and WS with the human cell-based assays (ERα-CALUX, ERα-GenBlazer, hERα-HeLa-9903 and MELN), while Fig. 3 shows those obtained with the yeast-based assay (LYES) and the non-cell-based assay (LiBERA). The tables below the plots include the EC_10_ (LYES, ERα-CALUX, hERα-HeLa-9903, MELN, ERα-GeneBLAzer) or IC_10_ (LiBERA) values in × EQS units (RM, HM, E2) or REF (WS) while the estrogenic potency, relative to E2, is expressed as EEQ in × EQS E2-Eq. The RM tested were Mix14, Mix19, Mix14NWL and Mix19NWL. All substances in the above-mentioned mixtures are present at 10,000 × EQS. Three WS were also tested, WS1 and WS2 are environmental samples, while WS-HM is an artificial sample reconstituted by spiking 1 L water with the HM (1000 × EQS) and enriched prior to bioanalyses as described in Section 2.1.1.

For each bioassay, RM and WS, dose-response curves were determined and EC_10_ and EC_50_ or IC_10_ and IC_50_ values (in the case of LiBERA) were interpolated or extrapolated from the curves. E2 was used as a reference to determine the estrogenic potency of the RM, HM and WS as described in the data evaluation section (Section 2.3). The EC_10_, EC_50_ or IC_50_ and IC_10_ values were used to determine the relative potency of the reference mixtures and WS to E2 by means of the EEQ (see Section 2.3).

EC_50_ values, EEQ in ng E2-Eq/L and BEQ of the WS relative to the different RM and HM are available in the supplementary information (Tables S3-S4). An EEQ value lower than 1 indicates a lower effect of the tested sample compared to the reference compound E2, while an EEQ value higher than 1 indicates higher effect of the tested sample compared to E2.

Differences in the responsiveness of the human cell-based bioassays (Fig. 2) to the different mixtures, WS and reference compound were observed. ERα-CALUX® (Fig. 2A) delivered similar EC_10_ values when testing E2, Mix14 (EEQ equal to 1 × EQS E2-Eq) and Mix19 (EEQ equal to 1.13 × EQS. E2-Eq). Mix14NWL elicited no effect and Mix19NWL had significantly lower effect than E2 (EEQ equal to 0.02 × EQS E2-Eq). The HM was more estrogenic than E2 (EEQ values higher than 1 × EQS E2-Eq). The three WS showed estrogenic effects, with WS2 being the most potent (EEQ equal to 4.92 × EQS E2-Eq).

ERα-GeneBLAzer (Fig. 2B) showed different EC_10_ values when testing E2, Mix14 (EEQ lower than 1 × EQS E2-Eq) and Mix19 (EEQ higher than 1 × EQS E2-Eq). Similar to ERα-CALUX®, Mix14NWL elicited no effect and Mix19NWL had significantly lower effect than E2 (EEQ equal to 0.28 × EQS E2-Eq). The HM was less estrogenic than E2 (EEQ equal to 0.46 × EQS E2-Eq). All the WS elicited estrogenic effects, confirming the WS2 as the most potent compared to E2 (EEQ equal to 6.68 × EQS E2-Eq).

EC_10_ values obtained in the hERα-HeLa-9903 assay (Fig. 2C) were slightly higher compared to E2 when testing Mix14 (EEQ equal to 0.68 × EQS E2-Eq), Mix19 (EEQ equal to 0.86 × EQS E2-Eq) and HM (EEQ equal to 0.72 × EQS E2-Eq). The curves of the aforementioned mixtures were similar to E2 as shown in Fig. 2C. Mix14NWL elicited no effect at the tested concentrations and Mix19NWL had significantly lower effect than E2 (EEQ equal to 0.12 × EQS E2-Eq). The three WS were estrogenic, being WS-HM the most potent compared to E2 (EEQ equal to 1.86 × EQS E2-Eq) followed by WS2 (EEQ equal to 1.83 × EQS E2-Eq).

MELN (Fig. 2D) showed higher EC_10_ values compared to E2 when testing Mix14 (EEQ equal to 0.80 × EQS E2-Eq) and Mix19 (EEQ equal to 0.83 × EQS E2-Eq). The EC_10_ value of HM was lower compared to E2 (EEQ equal to 8.25 × EQS E2-Eq). Mix14NWL elicited no effect and Mix19NWL had significantly lower effect than E2 (EEQ equal to 0.22 × EQS E2-Eq). The three WS were estrogenic, and the WS2 was the most potent compared to E2 (EEQ equal to 24.95 × EQS E2-Eq).

The yeast-based assay LYES delivered higher EC_10_ values compared to E2 when testing Mix14 (EEQ equal to 0.6 × EQS E2-Eq) and lower values when testing Mix19 (EEQ equal to 1.72 × EQS E2-Eq) (Fig. 3A). Mix14NWL elicited no effect and Mix19NWL had lower effect than E2 (EEQ equal to 0.55 × EQS E2-Eq). The EC_10_ value of HM was lower compared to E2 (EEQ equal to 1.41 × EQS E2-Eq). As in the other assays, the three WS were estrogenic with WS2 as the most potent compared to E2 (EEQ equal to 10.13 × EQS E2-Eq).

The non-cell-based assay LiBERA assay (Fig. 3B) responded differently with respect to the other assays, delivering similar IC_10_ values for all tested RM (Mix14, Mix19, Mix14NWL and Mix19NWL). Importantly, these RM were more estrogenic compared to E2 with the following values: Mix14 (EEQ equal to 61.94 × EQS E2-Eq), Mix19 (EEQ equal to 40.74 × EQS E2-Eq), Mix14NWL (EEQ equal to 35.16 × EQS E2-Eq) and Mix19NWL (EEQ equal to 53.59 × EQS E2-Eq). The IC_10_ value of HM was lower compared to E2 (EEQ equal to 2.05 × EQS E2-Eq). WS-HM was not tested in this bioassay, since when testing the HM at 10,000 × EQS we could not reach the 50% inhibitory effect. The two environmental WS were estrogenic, and also in this assay, the WS2 resulted the most potent compared to E2 (EEQ equal to 304.08 × EQS E2-Eq).

Table 3 shows a summary of the results obtained from testing the RM (Mix14, Mix19, Mix14NWL and Mix19NWL), HM, the two environmental WS (WS1 and WS2) and an artificial WS containing the three WL hormones (WS-HM). The estrogenic potency is expressed as EEQ in × EQS E2-Eq (3a) and ng/L E2-Eq (3b). An EEQ value higher than 1 × EQS E2-Eq or 0.4 ng/L E2-Eq indicates exceedance of estrogenic compounds in such mixture or sample, since 0.4 ng/L is the proposed safety limit concentration (EQS) for E2 established in the WL program under the WFD. Table 3 also includes the predicted mixture EEQ determined for the WS-HM (3c). For ERα-CALUX, hERα-HeLa9903 and MELN, the predicted EEQ values are lower than the experimental ones, indicating that these bioassays are able to detect the additive (or synergistic) effects of hormones.

**Table 3:** Experimental estrogenic potency of reference materials and water samples expressed as 17-β-estradiol (E2) equivalent concentration (EEQ) in × EQS. E2-Eq (a), ng/L E2-Eq (b), and (c) predicted EEQ of WS-HM in ng/L E2-Eq. The EEQ values are expressed as mean and standard deviation (SD) in brackets. HM, hormone mixture; Mix 14, mixture 14, Mix 19, mixture 19; Mix 14NWL, mixture 14 without watch list (WL) substances; Mix 19NWL, mixture 19 without watch list (WL) substances WS, water sample.

a)						
Mixture/Sample	(EEQ, EQS. E2-Eq)					
	ERα-CALUX	ERα-GeneBlazer	hERα-HeLa9903	MELN	LYES	LiBERA
Mix14	1.01(0.16)	0.74(0.03)	0.68(0.14)	0.80(0.22)	0.60(0.13)	61.9
Mix 19	1.13(0.13)	1.19(0.05)	0.86 (0.23)	0.83(0.07)	1.72(0.23)	40.7
Mix 14NWL	<0.01	<0.01	<0.01	<0.01	<0.01	35.2
Mix 19NWL	0.02	0.28(0.02)	0.12(0.13)	0.22(0.02)	0.55(0.04)	53.6
HM	1.41(0.21)	0.46(0.03)	0.72(0.71)	8.25(4.78)	1.41(0.18)	2.05
WS1	1.34(0.28)	1.68(0.08)	0.75(0.13)	3.62(0.88)	2.03(0.86)	184
WS2	4.92(0.45)	6.68(0.51)	1.83(2.05)	24.95 (3.43)	10.13(4.36)	304
WS-HM	3.14(0.93)	0.21(0.01)	1.86(1.91)	2.33(0.29)	0.90(0.29)	ND


b)						
Mixture/Sample	(EEQ, ng/L E2-Eq)					
	ERα-CALUX	ERα-GeneBlazer	hERα-HeLa9903	MELN	LYES	LiBERA
Mix14	0.41(0.06)	0.30(0.01)	0.27(0.06)	0.32 (0.08)	0.24(0.54)	24.8
Mix 19	0.45(0.05)	0.48(0.02)	0.34(0.09)	0.33(0.03)	0.69(0.09)	16.3
Mix 14NWL	< 0.01	< 0.01	< 0.01	< 0.01	< 0.01	14.1
Mix 19NWL	0.01	0.11(0.01)	0.05(0.05)	0.09(0.01)	0.22(0.02)	21.4
HM	0.56(0.09)	0.18(0.01)	0.29(0.28)	3.30(1.91)	0.57(0.07)	0.82
WS1	0.54(0.11)	0.68 (0.03)	0.21(0.05)	1.45(0.35)	0.81(0.35)	73.6
WS2	1.97(0.18)	2.67(0.20)	0.73(0.82)	9.98(1.37)	4.05(1.74)	121
WS-HM	1.25(0.37)	0.08(0.00)	0.74(0.76)	0.93(0.12)	0.36(0.12)	ND


c)						
Predicted EEQ WS-HM	ERα-CALUX	ERα-GeneBlazer	hERα-HeLa9903	MELN	LYES	LiBERA
(ng/L E2-Eq)	0.70	0.49	0.45	0.54	0.48	ND

The estrogenic effect of the WS (EC_50_ and EC_10_) was also compared with the estrogenic effect of the reference mixtures (Mix14, Mix19 and HM) through the respective BEQ (Mix14EQ, Mix19EQ and HMEQ). These results are summarized in the supplementary information (Tables S3 and S4). The estrogenic potency of WS2 expressed as EEQ, Mix14EQ and Mix19EQ was higher than 1 × EQS E2-Eq in all bioassays.

Moreover, for each bioassay, the WS concentration was expressed as × EQS by plotting the % effect value at 1 REF on the RM concentration-response curves. These results are shown in the supplementary information (Table S5 and Fig. S3). In all cases, the overall concentration of estrogens and/or xenoestrogens in WS2 exceeded the EQS value.

#### 3.2.1 Planar yeast estrogenic screen

**Fig. 4:**
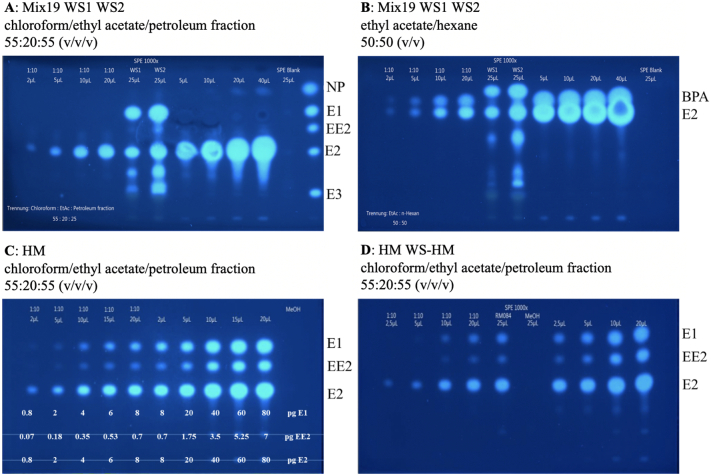
Separation of the mixture4 components by HPLC plates and analysis by p-YES. Volumes as indicated above were applied on the HPTLC plate. Panels A and B show the analysis of Mix19 and WS, panel C shows the dose-response relationship of HM, and panel D the characterization of WS-HM. 25 μL of a procedure blank in methanol were applied. Different volumes of Mix19 and HM were applied as indicated. The samples and standards were separated.

Mix19 without metals (Ni, Cd) and the WS (WS1, WS2) were analyzed by the planar yeast estrogenic screen (pYES) using two different mobile phases as shown in Fig. 4. E2 and BPA could be separated using ethyl-acetate/hexane 50:50 (*v*/v) and detected down to 0.02 times their respective amounts present in 1-L water at EQS-level indicating a sufficiently sensitive detection of these compounds taking into account a 1000-fold enrichment (Fig. 4).

Dose-response relationships for the estrogenic compounds E1, E2 and EE2 were generated using the HM 10,000 × EQS (Fig. S4). The analysis of HM 1000 × EQS showed a recovery of about 110% for the analytes with a relative standard deviation of around 25% (Table S7). Under consideration of a 1000-fold enrichment of the WS by SPE, the employed method is sensitive enough to detect the three WL compounds at EQS-level.

The WS showed a highly similar qualitative composition with the following candidate compounds: E1 and E2. WS2 additionally consisted of EE2 as well. The estrogenic potential of WS2 was about 2 to 3-fold stronger compared to WS1 (Table S8). Other unknown signals were observed during the analysis of the WS, two signals in WS1 accounting for 12% contribution to WS1 estrogenicity and three signals in WS2 contributing to 28% WS2 estrogenicity. One signal corresponding to the 5% estrogenicity might be assigned to estriol (E3) in both WS, while 7% and 22% of estrogenicity in WS1 and WS2, respectively, cannot be explained for any of the substances present in the RM (Table S9).

### 3.3 Gene expression analysis by quantitative real-time PCR

#### 3.3.1 Fish fathead minnow, exposures to fish early life stages

**Fig. 5:**
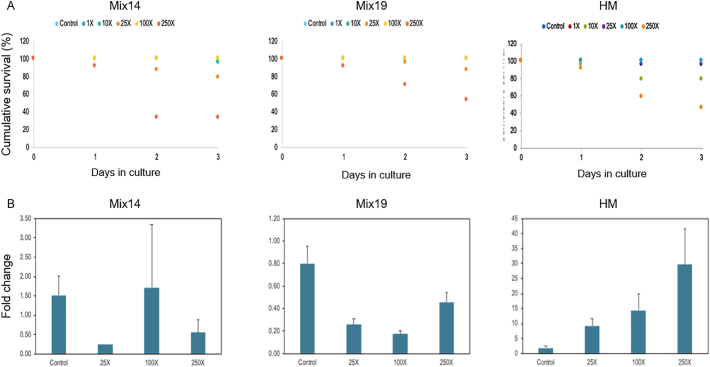
Vitellogenin (*Vtg*) expression in fathead minnow (*Pimephales promelas*) at early life stages after exposure to different EQS-fold concentrations of Mix14, Mix19 and HM. Results are expressed as fish cumulative survival (A) and fold change in *vtg* expression (B) respect to unexposed fish.

Mortality and *vtg* gene expression were studied *in vivo* using fathead minnow early life stage fish (Fig. 5A and 5B, respectively). No mortality was observed in the controls or the low concentration mixture solutions expressed as cumulative survival (Fig. 5A). However, mortality was observed at 25 × EQS and significant mortality at 250 × EQS concentrations. The 100 × EQS concentrations were less toxic than the 25 × EQS.

*Vtg* expression was used to monitor the estrogenic response in fish after exposure to the different chemical mixtures and measured by quantitative PCR (qPCR). As shown in Fig. 5B, exposure to the HM induced *vtg* expression in fish in a dose-responsive manner. However, the *vtg* response relative to the other two RM (Mix14 and Mix19) was not apparent, however a predominant anti-estrogenic effect was observed (Fig. 4B).

## 4 Discussion

The applicability of bioassays as screening methods to complement chemical analysis for water quality monitoring is currently being investigated given their potential advantages over chemical methods (Brack et al., 2019; Escher et al., 2014; Glauch and Escher, 2020; Neale et al., 2017; Wernersson et al., 2015). First, bioassays cover a broad range of modes of action (MoA). Second, they can account for additional risks posed by non-target compounds and chemical mixtures. Specific receptor-mediated MoAs, such as estrogenicity, are among the most relevant ones for water quality screening applications considering the widespread presence of endocrine disrupting compounds in the aquatic environment (Du et al., 2020; Escher et al., 2014; Leusch et al., 2018). However, it has been observed that bioassays covering estrogenic effects respond differently to specific estrogenic compounds (Carvalho et al., 2014; Kase et al., 2018; König et al., 2017; Kunz et al., 2017; Leusch et al., 2010), which resulted in the proposal to derive effect-based trigger values specific for each bioassays (Brion et al., 2019; Escher et al., 2018a; Jarošová et al., 2014a).

Although numerous studies have used estrogenicity assays on water samples, there are still open issues as to the potential of interaction with other components in defined “complex mixtures”. In this study, a reference complex mixture material was prepared to evaluate estrogenic effect detection against a defined background of other chemicals. These reference chemical mixture materials allowed detecting the combined effects (additive/synergistic/antagonistic) of multiple co-occurring chemicals in relation to EQS, taking into account the action of unknown substances which are not covered by standard chemical analytical methods.

Two of the reference materials were tested in a previous JRC EU-wide exercise (Carvalho et al., 2014) in a battery of ecotoxicity bioassays. The study demonstrated relevant effects of the artificial mixtures even when each substance in the mixture occurred at a concentration considered protective (EQS concentrations) for the environment. In this second exercise, the study was more focused on estrogenicity and to do so, we included an additional complex mixture composed of three estrogenic hormones in order to determine: *i)* whether the bioassays could detect the combined estrogenic effects of mixtures compared to E2; *ii)* if the bioassays could still be able to detect other low-affinity binding substances when the most potent compound (E2) was not present; and finally, *iii)* to assess the capability of the bioassays to detect estrogenic effects in environmental samples from areas impacted by distinct anthropogenic activities.

All *in vitro* bioassays (cell-based bioassays and LiBERA) were able to detect estrogenic effects of both environmental samples (WS1, WS2). None of the WL hormones (E1, E2 and EE2) or other possible estrogen agonists (*e.g.* NP, BPA) present in the mixtures was quantified in the WS when analyzed by chemical analysis, which is due to the LOQ of the analytical method as described previously (Könemann et al., 2018). Triclosan, a possible endocrine disruptor of very low specificity, was detected in WS1 at a concentration slightly exceeding the EQS. According to the literature, this substance seems not a ligand of the ER since it was found inactive in both human and zebrafish cell lines expressing the ER (Serra et al., 2018). However, it could interfere *in vivo* with sex hormone signaling pathways indirectly but the MoA is unknown. Of note, the majority of the substances present in the reference materials could not be quantified in the WS by chemical analysis since they were below the LOQ and far below their individual EQS. Nonetheless, the presence of E1 and E2 in both samples was confirmed by pYES which overall indicates that other unknown substances not covered by the RM and HM contributed to the estrogenicity of the WS. This was also evidenced by the cell-based bioassays, showing high EEQ values compared to the artificial WS (WS-HM), being MELN and LYES the ones that provided the highest EEQ values (10 and 25 × EQS E2-Eq, respectively). The relative estrogenic potency of WS2 compared to E2 was higher than the regulatory safety limit (EQS) established for this compound, indicating a relevant risk in the environment. When testing an artificial water sample (WS-HM) containing the WL hormones at EQS level, we could show that even at concentrations considered protective (EQS), the estrogenic response is triggered in some bioassays. The EEQ values delivered by ERα-CALUX, hERα-HeLa9903 and MELN were above the EQS and close to the EQS in the case of LYES indicating that the individual EQS may not be protective. Moreover, the experimental EEQ values of this sample were above the predicted mixture EEQ in ERα-CALUX, hERα-HeLa9903 and MELN, suggesting additive and possible synergistic effects. These findings confirm the conclusions of a previous study (Kase et al., 2018) indicating the need for effect-based screening methods to complement chemical analysis in the assessment of risk posed by mixtures of estrogenic compounds in the aquatic environment.

We determined the capability of each bioassay to detect estrogenic effects of mixtures in the presence and absence of hormones. In line with other studies (Brion et al., 2019; Kase et al., 2018; Kunz et al., 2017; Leusch et al., 2010; Schultis and Metzger, 2004), we observed differences in the responsiveness of the bioassays to the reference materials. Among the *in vitro* bioassays, the human cell-based assays were more responsive to E2, while the yeast-based assay was more responsive to Mix19. The non-cell-based assay (LiBERA) was more responsive to the Mix14, Mix19, Mix14NWL and Mix19NWL than to E2.

The observed differences among cell-based bioassays might be attributable, at least in part, to the specific cell contexts in which reporter gene systems are expressed and are influenced (*e.g.* permeability of cell-membrane, metabolic activities in the specific cell, differences in promoter sequence, expression level of ER). While LiBERA covers exclusively the binding affinity of the compounds (*i.e.*, including both agonists and antagonists) to the LBD of the hERα, the other bioassays are cellular reporter gene constructs, which constitute a more complex system involving further steps in the ER signaling pathway (*e.g.* uptake, binding to the ER, binding to specific DNA sequences and activation of the reporter gene expression). Dissimilarities in the responsiveness to the different mixtures were also observed among the cell-based assays which may be explained by differences in uptake and metabolism of substances depending on the cell type (toxicokinetic differences) but also differences in the receptor and reporter gene construct (toxicodynamics). Indeed, the yeast cell-based LYES assay showed lower sensitivity to E2 compared to the human cell-based assays, but it was able to catch the estrogenic effect of other substances in the mixture when E2 was not present. LYES provided the highest EEQ value for Mix19NWL (0.55 × EQS E2-Eq). Metabolic capacities, membrane permeability (for the YES), different transcriptional cofactors may contribute more to the between-assays differences in response to xenoestrogens (other than steroids).

Among the human cell-based assays, ERα-CALUX® was the most sensitive to E2 as already reported by Könemann et al. (2018). The response of this bioassay to Mix14, Mix19 and E2 was similar, but no estrogenic effects of other mixture compounds were detected when E2 was not present. This was observed when testing Mix14NWL and Mix19NWL, which did not contain E2. Mix14NWL had no effect in this bioassay and the effect of Mix19NWL was very low compared to E2 (EEQ equal to 0.02 × EQS E2-Eq). The results suggest that ERα-CALUX® might not be able to detect the combined effects of other substances present in complex mixtures and confirms E2 as the main driver of estrogenicity. When using HM to understand whether the bioassays were able to detect the additive effects of the three WL hormones, MELN proved to be the most sensitive (EEQ value was 8.25 × EQS E2-Eq) among employed methods, while ERα-CALUX® showed lower response to this reference material but still it delivered EC_10_ values below pure E2 suggesting that a possible additive effect of estrogens may be detected by this bioassay. However, detection of additive effects by the two bioassays may be influenced by different estrogens present in a sample. According to the literature, ERα-CALUX® is more sensitive to E2 and EE2, while MELN is the most sensitive to E1 (Könemann et al., 2018). Typically, the MELN cell line is based on MCF-7 cells which are able to metabolize E1 into E2 making E1 more active in MELN (REP 0.2) than in other assays developed in more “neutral” cell systems (*e.g.* REP 0.02 in ERα-CALUX® or hERα-HeLa-9903), hence leading to higher E2-Eqs in MELN. MELN is the only bioassay in this study based on human cells endogenously expressing the ER, which due to their metabolic capacities and endogenous expression of steroidogenesis enzymes could contribute to modulate its sensitivity to steroid estrogens. This could explain the highest response of this bioassay to the estrogenic hormones and thus its ability to detect mixture effects of HM. The hER expression level in the different human cell lines is an additional critical point that could contribute to the observed differences in responsiveness and, upon saturating hormone concentrations, lead to the loss of sensitivity.

When comparing the overall outcomes of the *in vitro* and *in vivo* bioassays, we identified differences between responses to the RM and HM, since *in vitro* bioassays cover only part of the metabolic process occurring in the organisms which may lead to false positive or false negative results. According to the literature*, in vitro* bioassays display similar sensitivity to E2 and EE2, and lower to E1, while the *in vivo* bioassays show similar responsiveness to E1 and E2 (Könemann et al., 2018) but higher response to EE2 (Brion et al., 2012). These differences can be explained by the higher stability of EE2 with respect to E1 and E2 which might prevent it from being degraded or metabolized. Our results in fathead minnow fish showed a predominant anti-estrogenic effect of chemical mixtures (Mix14, Mix19) compared to the HM. The decrease in *vtg* expression could be explained by the presence of ER antagonists in the tested mixtures, or substances interacting with other related receptors such as the aryl hydrocarbon receptor (AhR). For example, AhR is a binding target of PAHs and a cross-talk between ER and AhR signaling pathways has been described in fish hepatocytes (Bemanian et al., 2004). Possibly, the PAHs (*e.g.* BaP, fluoranthene) present in the RM (Mix14, Mix19, Mix14NWL and Mix19NWL) inhibit the expression of *vtg* and *erα* genes by acting through the AhR. This is very likely to happen for BaP but not for fluoranthene, which did not induce AhR response in PLHC-1 cells (Louiz et al., 2008) and resulted a very weak AhR activator in PAH-CALUX (Boonen et al., 2020). Regarding the ER-AhR cross-talk mechanism, it also occurs in the cell systems used in this study as AhR is expressed in most of the cell lines. In MELN for instance, AhR ligands (BaP, TCDD) will increase ER-luciferase expression through a positive cross-talk (Balaguer et al., 1999; Serra et al., 2019). In HeLa cells, a negative cross-talk is present (*i.e.*, inhibition of ER-luciferase by TCDD) (Balaguer et al., 1999). However, such anti-estrogenic effect was not observed in the cell-based estrogenicity bioassays carried out in this study. The RM effects were also studied in *Poeciliopsis lucida* hepatocellular carcinoma-derived PLHC-1 cell line (Fig. S4) in which expression of the *erα* and *vtg* biomarkers was induced at high concentrations by Mix14, Mix14NWL and Mix19NWL but not stimulated by the Mix19 or HM.

The use of reference materials provided information to which extent detection of estrogenicity effects varies throughout a panel of *in vitro* and *in vivo* bioassays showing differences in their response (Table 3) and permitted to identify either the advantages or possible methodological drawbacks. *In vivo* and *in vitro* bioassays performed differently but both are valuable for providing information on different effects in WS. Moreover, our results indicate that some of the bioassays routinely used in water quality monitoring do not cover all substances with potential endocrine disrupting properties present in water. For instance, the estrogenic response in ERα-CALUX® is mainly triggered by E2 and other potent estrogens, such as EE2, underestimating the contribution of other estrogenic substances, such as BPA. Therefore, complementary bioassays, such as pYES or LiBERA, with an enhanced responsiveness to substances other than estrogenic hormones, would provide additional information. Rather than selecting one bioassay, it would be recommended to use a set of bioassays with different responsiveness providing an integrated measure of the active toxicants present in an environmental sample. Such differences need to be taken into account when a single bioassay is selected for monitoring activities. In this sense, a specific bioassay (or a set of assays) could be selected and adapted to specific environmental pollution conditions (*e.g.* wastewater effluents, surface water from urban or agricultural areas) to optimize cost/benefit. Indeed, the choice of the bioassays could be determined by the degree of source pollution. Overall, a highly polluted area such as wastewater effluent would not require a very sensitive bioassay comparing to a surface water, far distant from a treatment plant, thus rationalizing the cost.

Finally, RM allowed linking EQS to biological effects. Nevertheless, the selection of substances to constitute the RM should be further investigated in order to establish the most appropriate mixture composition for the effects to be assessed. Furthermore, before analyzing emerging substances in mixture, it would be preferable first to test one by one in order to better define their effects and then endpoints in the bioassay. Creation of tailored RM for specific environmental sites could serve as the basis to perform surface water pollution profiling.

## 5 Conclusion

Chemical mixtures, which are ubiquitously present in waterbodies, negatively impact water quality. This is particularly the case for the estrogenicity/and EDC mixture because their effects may be elicited at very low concentrations, threatening the life of aquatic organisms and indirectly many other organisms including humans. In this study, we showed the powerful combination of *in vitro* and *in vivo* bioassays as well as their ability to detect the estrogenic effects even when the substances could not be detected by the chemical analysis, as the case for WS2. These bioassays, although some of them, routinely used in the laboratories by local authorities, are ready to be implemented for assessing the estrogenicity/EDC mixture in a regulatory process to enhance the water quality protection at the European and global level. To achieve this goal, in Europe the Member States must be engaged in the validation of SOP, in training to handle the bioassays and in performing the monitoring under a European framework (*e.g.* the Watch List program under the WFD). The bioassays could be, then, included, either as an alternative method or complementary to the chemical analysis (*e.g.* in the WFD), as well for testing the efficiency removal of a wastewater treatment plant (WWTP), for reducing the release of estrogens/EDC in surface waters and for water reuse.

Furthermore, existing and ready-to-use bioassay are not exclusively to the estrogenicity assays since others (*e.g.* algae test, Ames test for herbicide and mutagenicity endpoint, respectively) could be also implemented as first glance, to cover the mixture effects of other pollutants to better protect the aquatic ecosystem.

## Funding

RECETOX authors were supported by RECETOX Research Infrastructure grant LM2018121 from the Czech 10.13039/501100001823Ministry of Education, Youth and Sports; the platform CITEPro (Chemicals in the Environment Profiler) funded by the 10.13039/501100001656Helmholtz Association for performing the ER-GeneBLAZer assay; INERIS authors were supported by the French 10.13039/501100003959Ministry of Ecology (P181-DRC60) and AQUAREF.

## CRediT authorship contribution statement

**Livia Gómez:** Investigation, Formal analysis, Data curation, Visualization, Writing – original draft, Writing – review & editing. **Magdalena Niegowska:** Data curation, Visualization, Writing – original draft, Writing – review & editing. **Anna Navarro:** Investigation, Formal analysis, Writing – review & editing. **Luca Amendola:** Investigation, Formal analysis, Writing – review & editing. **Augustine Arukwe:** Investigation, Formal analysis, Writing – review & editing. **Selim Ait-Aissa:** Investigation, Formal analysis, Writing – review & editing. **Stefania Balzamo:** Writing – review & editing. **Salvatore Barreca:** Investigation, Formal analysis, Writing – review & editing. **Shimshon Belkin:** Writing – review & editing. **Michal Bittner:** Investigation, Formal analysis, Writing – review & editing. **Ludek Blaha:** Investigation, Formal analysis, Writing – review & editing. **Sebastian Buchinger:** Investigation, Formal analysis, Writing – review & editing. **Maddalena Busetto:** Investigation, Formal analysis, Writing – review & editing. **Mario Carere:** Writing – review & editing. **Luisa Colzani:** Investigation, Formal analysis, Writing – review & editing. **Pierluisa Dellavedova:** Investigation, Formal analysis, Writing – review & editing. **Nancy Denslow:** Investigation, Formal analysis, Writing – review & editing. **Beate I. Escher:** Investigation, Formal analysis, Writing – review & editing. **Christer Hogstrand:** Writing – review & editing. **Essa Ahsan Khan:** Investigation, Formal analysis, Writing – review & editing. **Maria König:** Investigation, Formal analysis, Writing – review & editing. **Kevin J. Kroll:** Investigation, Formal analysis, Writing – review & editing. **Ines Lacchetti:** Writing – review & editing. **Emmanuelle Maillot-Marechal:** Investigation, Formal analysis, Writing – review & editing. **Liat Moscovici:** Writing – review & editing. **Monica Potalivo:** Investigation, Formal analysis, Writing – review & editing. **Isabella Sanseverino:** Writing – review & editing. **Ricardo Santos:** Writing – review & editing. **Andrea Schifferli:** Investigation, Formal analysis, Writing – review & editing. **Rita Schlichting:** Investigation, Formal analysis, Writing – review & editing. **Susanna Sforzini:** Writing – review & editing. **Eszter Simon:** Investigation, Formal analysis, Writing – review & editing. **Etai Shpigel:** Writing – review & editing. **Stephen Sturzenbaum:** Writing – review & editing. **Etienne Vermeirssen:** Investigation, Formal analysis, Writing – review & editing. **Aldo Viarengo:** Writing – review & editing. **Inge Werner:** Writing – review & editing. **Teresa Lettieri:** Conceptualization, Writing – original draft, Writing – review & editing.

## Declaration of competing interest

The authors declare that they have no known competing financial interests or personal relationships that could have appeared to influence the work reported in this paper.

## Appendix A Supplementary data

Supplementary information Image 1
