# Supplementary material for: Estrogenicity of chemical mixtures revealed by a panel of bioassays
Source: Sci Total Environ. 2021 Sep 1;785:147284. doi: 10.1016/j.scitotenv.2021.147284 (PMC8210648; doi:10.1016/j.scitotenv.2021.147284)
Supplement: Supplementary file 1 — Supplementary information [file mmc1.docx]

Estrogenicity of chemical mixtures revealed by a panel of bioassays

Livia Gómez^a^, Magdalena Niegowska^a^, Anna Navarro^a^, Luca Amendola^b^, Augustine Arukwe^c^, Selim Ait-Aissa^d^, Stefania Balzamo^e^, Salvatore Barreca^f^ ,Shimshon Belkin^g^, Michal Bittner^h^, Ludek Blaha^h^, Sebastian Buchinger^i^, Maddalena Busetto^f^, Mario Carere^j^,, Luisa Colzani^f^, Pierluisa Dellavedova^f^, Nancy Denslow^k^, Beate I. Escher^l^, Christer Hogstrand^m^, Essa Ahsan Khan^c^, Maria König^l^, Kevin J. Kroll^k^, Ines Lacchetti ^j^, Emmanuelle Maillot-Marechal^d^, Liat Moscovici^g^, Monica Potalivo^e^, Isabella Sanseverino^a^, Ricardo Santos^n^, Andrea Schifferli^o^, Rita Schlichting^l^, Susanna Sforzini^p^, Eszter Simon^o,q^, Etai Shpigel^g^, Stephen Sturzenbaum^r^, Etienne Vermeirssen^o^, Aldo Viarengo^p^, Inge Werner^o^ and Teresa Lettieri^a*^

^a^ European Commission, Joint Research Centre (JRC), Via E. Fermi 2749, 21027 Ispra (VA), Italy.

^b^ ARPA Lazio, Regional Agency for Environmental Protection, Via G. Saredo 52, 00173 Rome, Italy.

^c^ Department of Biology, Norwegian University of Science and Technology (NTNU), NO-7491 Trondheim, Norway.

^d^ French National Institute for Industrial Environment and Risks (INERIS), UMR-I 02 SEBIO, 60550 Verneuil-en-Halatte, France.

^e^ ISPRA - Environmental Metrology Unit, Via di Castel Romano 100, 00128 Rome, Italy.

^f^ ARPA Lombardia, Regional Agency for Environmental Protection, Via Rosellini 17, 20124 Milan, Italy.

^g^ Department of Plant and Environmental Sciences, The Alexander Silberman Institute of Life Sciences, The Hebrew University of Jerusalem, Jerusalem 91904, Israel.

^h^ RECETOX, Faculty of Science, Masaryk University, Kamenice 5, CZ62500 Brno, Czech Republic.

^i^ Federal Institute of Hydrology, Am Mainzer Tor 1, D-56068 Koblenz, Germany.

^j^ ISS-National Health Institute, Viale Regina Elena 299, 00161 Rome, Italy.

^k^ Center for Environmental & Human Toxicology, Department of Physiological Sciences, College of Veterinary Medicine, University of Florida, Gainesville, FL, USA; University of Florida Genetics Institute, USA.

^l^ Department Cell Toxicology, Helmholtz Centre for Environmental Research - UFZ, Permoserstraße 15, 04318 Leipzig, Germany.

^m^ Metal Metabolism Group, Department of Nutritional Sciences, Faculty of Life Sciences and Medicine, King's College London, 150 Stamford St, London SE1 9NH, UK.

^n^ Laboratório de Análises, Instituto Superior Tecnico, Universidade de Lisboa, Av. Rovisco Pais, 1049-001 Lisboa, Portugal.

^o^ Swiss Centre for Applied Ecotoxicology, Überlandstrasse 133, 8600, Dübendorf, Switzerland.

^p^ Institute for the Study of Anthropic Impacts and Sustainability in Marine Environment, National Research Council (CNR-IAS) Via de Marini 6, Genova 16149, Italy.

^q^ Present address: Federal Ofﬁce for the Environment FOEN, Air Pollution Control and Chemicals Division, Industrial Chemical Section, CH-3003 Bern, Switzerland.

^r^ School of Population Health & Environmental Sciences, Faculty of Life Sciences & Medicine, King's College London, 150 Stamford Street, London, U.K.

^*^ Corresponding author:

E-mail: [teresa.lettieri@ec.europa.eu](mailto:teresa.lettieri@ec.europa.eu)

Postal address: Via E. Fermi 2749, 21027 Ispra (VA), Italy.

**Supplementary Information**

**SUMMARY**

**Supplementary Tables**

| Table S1 | page 5 |
| --- | --- |
| Table S2 | page 6 |
| Table S3 | page 7 |
| Table S4 | page 9 |
| Table S5 | page 11 |
| Table S6 | page 13 |
| Table S7 | page 13 |
| Table S8 | page 13 |
| Table S9 | page 14 |
| Table S10 | page 23 |
| Table S11 | page 23 |
| Table S12 | page 24 |
| Table S13 | page 24 |
| Table S14 | page 24 |
| Table S15 | page 27 |
| Table S16 | page 30 |
| Table S17 | page 31 |
| Table S18 | page 33 |
| Table S19 | page 35 |
| Table S20 | page 36 |
| Table S21 | page 38 |
| Table S22 | page 39 |
| Table S23 | page 40 |
| Table S24 | page 40 |

**Supplementary Figures**

| Figure S1 | page 15 |
| --- | --- |
| Figure S2 | page 16 |
| Figure S3 | page 18 |
| Figure S4 | page 19 |
| Figure S5 | page 20 |
| Figure S6 | page 26 |
| Figure S7 | page 29 |
| Figure S8 | page 32 |
| Figure S9 | page 34 |
| Figure S10  Figure S11 | page 36  page 37 |

**Supplementary Materials and Methods**

| Composition of Reference Mixtures | page 22 |
| --- | --- |
| Stability and homogeneity studies | page 25 |
| Analytical methods for water samples  Cytotoxicity of reference mixtures and water samples | page 39  page 41 |

**Supplementary references**

|  | page 42 |
| --- | --- |

**Supplementary tables**

TABLE S1. List of bioassays and partner laboratories in the EU-wide estrogenicity study. The JRC led a consortium of eight research Institutes from seven countries in EU, US and Switzerland to assess the application of reference materials (e.g. RM, HM) to determine the water quality profile of environmental and artificial water samples (e.g. WS, WS-HM).

|  | **Partner**  **Laboratory^a^**  **Organism**  **and/or test** | | **JRC (EC)** | | **ECOTOX (CH)** | | **RECETOX (CZ)** | | **INERIS (FR)** | | **NTNU (NO)** | | **CEHT (USA)** | | **UFZ (DE)** | | **BFG (DE)** | |
| --- | --- | --- | --- | --- | --- | --- | --- | --- | --- | --- | --- | --- | --- | --- | --- | --- | --- | --- |
| ***In vitro* human and yeast cell-based estrogenicity bioassays** | Lyticase Yeast estrogenic screen (LYES) |  | | X | |  | |  | |  | |  | |  | |  | |  |
|  | ERα-CALUX^®^ |  | | X | |  | |  | |  | |  | |  | |  | |  |
|  | ERα-HeLa-9903 cell line  (estrogenicity) |  | |  | | X | |  | |  | |  | |  | |  | |  |
|  | ERα-HeLa-9903 cell line  (anti-estrogenicity) |  | |  | | X | |  | |  | |  | |  | |  | |  |
|  | ER-binding/activation in MELN cells |  | |  | |  | | X | |  | |  | |  | |  | |  |
|  | ERα GeneBLAzer |  | |  | |  | |  | |  | |  | | X | |  | |  |
|  | Planar Yeast estrogenic screen (pYES) |  | |  | |  | |  | |  | |  | |  | | X | |  |
| **Receptor binding** | Ligand Binding Estrogen Receptor Assay (LiBERA) | X | |  | |  | |  | |  | |  | |  | |  | |  |
| **Gene expression analysis with qRT-PCR** | *Poeciliopsis lucida* hepatocellular carcinoma derived PLHC-1 cell line exposure biomarkers, erα and vtg |  | |  | |  | |  | | X | |  | |  | |  | |  |
|  | Fish Fathead minnow  Exposures to fish early life stages, *vtg* biomarker |  | |  | |  | |  | |  | | X | |  | |  | |  |

^a^ The following abbreviations have been used for the different partner laboratories, in alphabetical order:

BFG, Federal Institute of Hydrology, Am Mainzer Tor 1, D-56068 Koblenz, Germany

DTU, Technical University of Denmark (Department of Environmental Engineering), Kgs Lyngby, Denmark

CEHT, Center for Environmental & Human Toxicology, Department of Physiological Sciences, College of Veterinary Medicine, University of Florida, Gainesville, FL, USA; University of Florida Genetics Institute, USA

ECOTOX, Swiss Centre for Applied Ecotoxicology, Dübendorf, Switzerland

INERIS, National Institute for Environmental Technology and Hazards, Verneuil en Halatte, France

JRC, European Commission - Joint Research Centre, Ispra, Italy

NTNU, Norwegian University of Science & Technology (NTNU), Trondheim, NORWAY

RECETOX, Research Centre for Toxic Compounds in the Environment, Masaryk University, Brno, Czech Republic

UFZ, Helmholtz Centre for Environmental Research - Department Cell Toxicology, Permoserstraße 15, 04318 Leipzig, Germany

TABLE S2. Primer pair sequences with annealing temperature conditions for genes quantified with real-time PCR*.*

| **Genes** | **Primers** | **Sequence** | **Annealing temperature (°C)** |
| --- | --- | --- | --- |
| ***erα*** | Forward | ATTCTGTGCTCCCGTCAGAA | 60 |
|  | Reverse | CTTCTTTGCCCAAGCGATCA |  |
| ***vtg*** | Forward | AGCTTTGCTCACTCCTGGAT | 60 |
|  | Reverse | GGGTGGTCTTGATTGGCAAG |  |

TABLE S3. Summary of the human cell-based bioassay results after exposure to the mixtures (Mix14, Mix19, Mix14NWL, Mix19NWL, HM) and water samples (WS-HM, WS1, WS2). EC_10_ and EC_50_ values are expressed as ng/L and EQS multiple values (x EQS). The EEQ values are expressed as ng/L E2-equivalents and x EQS E2--equivalents. LOQ, limit of quantification; NE, no effect. The values are presented as mean with the standard deviation (SD) in brackets except for ER-GeneBLAzer for which the standard error is provided (SE) in brackets. For WS1 and WS2, also other BEQ values are presented relative to Mix14 (x EQS Mix14-equivalents), Mix19 (x EQS Mix19-equivalents) and HM (x EQS HM-equivalents) effect.

| ***Bioassay***  ***Sample*** | | ***ERα-CALUX*** | | ***ERα-GeneBLAzer*** | ***ERα-HeLa-9903*** | | ***MELN*** | |
| --- | --- | --- | --- | --- | --- | --- | --- | --- |
|  |  | **EC_10_** | **EC_50_** | **EC_10_** | **EC_10_** | **EC_50_** | **EC_10_** | **EC_50_** |
| **E2** | **ng/L**  **x EQS** | 0.38(0.02)  0.95(0.06) | 1.45(0.12)  3.62(0.30) | 5.81(0.15)  14.5(0.38) | 1.7  4.33 | 13.7  34.4 | 1.25(0.17)  3.15(0.43) | 4.18(0.97)  10.46(2.43) |
| **MIX 14** | **x EQS**  **EEQ (ng/L)**  **EEQ (x EQS)** | 0.96(0.16)  0.41(0.06)  1.01(0.16) | 4.90(0.68)  0.30(0.05)  0.75(0.08) | 19.37(0.72)  0.30(0.01)  0.74(0.03) | 6.33(1.00)  0.27(0.06)  0.68(0.14) | 34.38(12.9)  0.40(0.15)  1.00(0.38) | 4.18(1.49)  0.32 (0.08)  0.80(0.22) | 16.58(4.5)  0.26(0.06)  0.65(0.15) |
| **MIX 19** | **x EQS**  **EEQ (ng/L)**  **EEQ (x EQS)** | 0.85(0.10)  0.45(0.05)  1.13(0.13) | 4.25(0.9)  0.35(0.08)  0.88(0.21) | 12.16(0.39)  0.48(0.02)  1.19(0.05) | 5.03(0.96)  0.34(0.09)  0.86 (0.23) | 35.70(9.65)  0.39(0.15)  0.960.37) | 3.78(0.25)  0.33(0.03)  0.83(0.07) | 15.14(4.72)  0.29(0.09)  0.73(0.23) |
| **MIX 14 NWL** | **x EQS**  **EEQ (ng/L)**  **EEQ (x EQS)** | <LOQ | <LOQ | <10% | <10% | <10% | <10% | <10% |
| **MIX 19 NWL** | **x EQS**  **EEQ (ng/L)**  **EEQ (x EQS)** | 38.3  =LOQ(0.01)  0.02 | 331.5  =LOQ(0.01)  0.01 | 52.14(2.85)  0.11(0.01)  0.28(0.02) | 36.21(16.54)  0.05(0.05)  0.12(0.13) | <50% | 14.02(1.40)  0.09(0.01)  0.22(0.02) | <50% |
| **HM** | **x EQS**  **EEQ (ng/L)**  **EEQ (x EQS)** | 0.69(0.10)  0.56(0.09)  1.41(0.21) | 3.26(0.26)  0.45(0.04)  1.12(0.09) | 32.58(1.82)  0.18(0.01)  0.46(0.03) | 6.06(2.83)  0.29(0.28)  0.72(0.71) | 36.86(7.74)  0.37(0.05)  0.93(0.13) | 0.46(0.22)  3.30(1.91)  8.25(4.78) | 4.62(0.12)  091(0.23)  2.27(0.57) |
| **WS1** | **REF**  **EEQ (ng/L)**  **EEQ x EQS**  **MIX14EQ**  **MIX19EQ**  **HMEQ** | 0.73(0.17)  0.54(0.11)  1.34(0.28)  1.35(0.28)  1.20(0.25)  0.97(0.2) | 4.33(0.45)  0.34(0.03)  0.84(0.08)  1.14(0.11)  0.99(0.1)  0.76(0.07) | 6.57(0.31)  0.68 (0.03)  1.68(0.08)  2.26(0.66)  1.42(0.39)  3.80(1.37) | 8.42(1.52)  0.21(0.05)  0.75(0.13)  0.69(0.13)  0.55(0.03)  0.72(1.86) | <50% | 0.90(0.24)  1.45(0.35)  3.62(0.88)  3.45(1.47)  4.94(2.10)  0.29(0.12) | 5.54(1.62)  0.78(0.19)  1.96(0.48)  2.67(0.69)  2.65(0.69)  0.98(0.25) |
| **WS2** | **REF**  **EEQ (ng/L)**  **EEQ x EQS**  **MIX14EQ**  **MIX19EQ**  **HMEQ** | 0.20(0.02)  1.97(0.18)  4.92(0.45)  4.94(0.46)  4.40(0.41)  3.54(0.33) | 0.75(0.26)  2.13(0.88)  5.33(2.20)  7.22(2.98)  6.27(2.58)  4.80(1.98) | 2.18(0.15)  2.67(0.20)  6.68(0.51)  8.90(0.24)  5.59(0.15)  14.98(0.46) | 2.36(1.63)  0.73(0.82)  1.83(2.05)  2.68(1.83)  2.13(1.22)  2.56(1.74) | <50% | 0.13(0.01)  9.98(1.37)  24.95 (3.43)  14.56(3.85)  20.85(5.52)  1.21(0.32) | 0.75(0.17)  5.57(0.09)  13.93(0.24)  12.03(2.63)  11.99(2.62)  4.41(0.96) |
| **WS-HM** | **x EQS (REF)**  **EEQ (ng/L)**  **EEQ (x EQS)**  **HMEQ** | 0.32(0.09)  1.25(0.37)  3.14(0.93)  2.16(1.11) | 3.03(1.74)  0.68(0.55)  1.71(1.37)  1.08(0.15) | 70.77(3.76)  0.08(0.00)  0.21(0.01)  0.46(0.50) | 2.33(1.15)  0.74(0.76)  1.86(1.91)  2.60(2.10) | 16.82(1.97)  0.81(0.12)  2.50(0.29)  2.19(1.92) | 1.36 (0.17)  0.93(0.12)  2.33(0.29)  0.30(0.06) | 5.17(1.41)  0.84(0.29)  2.12(0.73)  0.89(0.10) |

TABLE S4. Summary of the yeast cell-based bioassay (LYES) and non-cell-based assay (LiBERA) results after exposure to the mixtures (Mix14, Mix19, Mix14NWL, Mix19NWL, HM) and water samples (WS-HM, WS1, WS2). EC_10_ or IC_10_ and EC_50_ or IC_50_ values are expressed as ng/L and EQS multiple values (x EQS). The EEQ values are expressed as ng/L E2-equivalents and x EQS E2-equivalents. LOQ, limit of quantification; ND, not determined. For WS1 and WS2 also other BEQ values are presented relative to Mix14 (x EQS Mix14-equivalents), Mix19 (x EQS Mix19-equivalents) and HM (x EQS HM-equivalents) effect.

| ***Bioassay***  ***Sample*** | | ***LYES*** | | ***LiBERA*** | |
| --- | --- | --- | --- | --- | --- |
|  |  | **EC_10_** | **EC_50_** | **IC_10_** | **IC_50_** |
| **E2** | **ng/L**  **x EQS** | 8.6(2.1)  21.62(5.28) | 28.8(8.9)  71.89(22.3) | 670.27  1675.69 | 4064.99  10162.49 |
| **MIX 14** | **x EQS**  **EEQ (ng/L)**  **EEQ (x EQS)** | 37.2(8.3)  0.24(0.54)  0.60(0.13) | 125.1(44.1)  0.25(0.07)  0.62(0.18) | 27.05  24.77  61.99 | 164.06  24.77  61.99 |
| **MIX 19** | **x EQS**  **EEQ (ng/L)**  **EEQ (x EQS)** | 12.7(1.8)  0.69(0.09)  1.72(0.23) | 42.6(11.2)  0.71(0.18)  1.76(0.44) | 41.13  14.06  40.74 | 249.46  14.06  40.7 |
| **MIX 14 NWL** | **x EQS**  **EEQ (ng/L)**  **EEQ (x EQS)** | <LOQ | <LOQ | 47.66  14.06  35.15 | 289.07  14.06  35.15 |
| **MIX 19 NWL** | **x EQS**  **EEQ (ng/L)**  **EEQ (x EQS)** | 39.3(2.9)  0.22(0.02)  0.55(0.04) | cytotoxicity | 31.27  21.43  53.57 | 189.67  21.43  53.57 |
| **HM** | **x EQS**  **EEQ (ng/L)**  **EEQ (xEQS)** | 15.5(2.1)  0.57(0.07)  1.41(0.18) | 45.2(4.9)  0.64(0.07)  1.60(0.17) | 816.94  0.82  2.05 | <50% |
| **WS1** | **REF**  **EEQ (ng/L)**  **EEQ x EQS**  **MIX14EQ**  **MIX19EQ**  **HMEQ** | 12.7(7.2)  0.81(0.35)  2.03(0.86)  3.48(1.49)  1.19(0.51)  1.45(0.62) | 72.6(15.3)  0.41(0.09)  1.01(0.21)  1.76(0.37)  0.59(0.13)  0.64(0.13) | 9.10  73.63  184.07  2.97  4.51  89.74 | <50% |
| **WS2** | **REF**  **EEQ (ng/L)**  **EEQ x EQS**  **MIX14EQ**  **MIX19EQ**  **HMEQ** | 2.55(1.46)  4.05(1.74)  10.13(4.36)  17.41(7.49)  5.95(2.56)  7.25(3.12) | 10.3(5.6)  3.27(1.34)  8.17(3.35)  14.22(5.83)  4.84(1.99)  5.13(2.11) | 5.51  121.63  304.08  4.99  7.46  148.25 | <50% |
| **WS-HM** | **x EQS**  **EEQ (ng/L)**  **EEQ (x EQS)**  **HMEQ** | 26.2(10.5)  0.36(0.12)  0.60(0.29)  0.58(0.2) | 87.9(45.7)  0.38(0.15)  0.51(0.38)  0.51(0.12) | N.D. | N.D. |

TABLE S5.

Extrapolated effect-concentration values of the water samples (WS1, WS2 and WS-HM) at 1 REF to the different mixtures (Mix14, Mix19, Mix14 NWL, Mix19 NWL and HM). ND; not determined. The concentration-response curves prepared for the WS were used to determine the % of effect corresponding to a concentration 1 REF (not diluted or concentrated) in each bioassay. These values were then used to extrapolate the corresponding effect-concentration in the concentration-response curves prepared with the reference materials (calibration curves).

| **WS1** | | **Extrapolated effect concentration (x EQS) at 1 REF** | | | | | |
| --- | --- | --- | --- | --- | --- | --- | --- |
|  |  | **E2** | **Mix14** | **Mix19** | **Mix14 NWL** | **Mix19 NWL** | **HM** |
| **Bioassay** | **ER*α*-CALUX** | 1.195 | 1.332 | 1.301 | - | 36295.789 | 0.357 |
|  | **Er*α-*GenBLAzer** | 0.048 | 0.144 | 0.154 | - | 0.852 | 0.319 |
|  | **ERα*-*HeLa-9903** | 0.905 | 1.762 | 1.057 | - | 12.104 | 1.549 |
|  | **MELN** | 4.101 | 4.351 | 4.263 | - | 16.013 | 0.526 |
|  | **LYES** | 11.727 | 22.391 | 6.886 | - | 17.404 | 8.879 |
|  | **LiBERA** | 184.077 | 2.972 | 4.519 | 5.236 | 3.436 | 89.743 |

| **WS2** | | **Extrapolated effect concentration (x EQS) at 1 REF** | | | | | |
| --- | --- | --- | --- | --- | --- | --- | --- |
|  |  | **E2** | **Mix14** | **Mix19** | **Mix14 NWL** | **Mix19 NWL** | **HM** |
| **Bioassay** | **ER*α*-CALUX** | 6.203 | 8.061 | 6.085 | - | 3326960815484.250 | 5.307 |
|  | **ER*α*-GenBLAzer** | 1.068 | 2.998 | 2.566 | - | 16.723 | 5.656 |
|  | **ERα-HeLa-9903** | 2.420 | 3.934 | 2.739 | - | 24.103 | 3.652 |
|  | **MELN** | 15.370 | 19.198 | 16.513 | - | 110.468 | 6.703 |
|  | **LYES** | 17.903 | 35.911 | 11.564 | - | 37.510 | 14.200 |
|  | **LiBERA** | 304.089 | 4.909 | 7.464 | 8.650 | 5.675 | 148.252 |

| **WS-HM** | | **Extrapolated effect concentration (x EQS) at 1 REF** | | | | | |
| --- | --- | --- | --- | --- | --- | --- | --- |
|  |  | **E2** | **Mix14** | **Mix19** | **Mix14 NWL** | **Mix19 NWL** | **HM** |
| **Bioassay** | **ER*α*-CALUX** | 2.136 | 2.513 | 2.242 | - | 23352371.758 | 1.745 |
|  | **ER*α*-GenBLAzer** | 0.221 | 0.640 | 0.614 | - | 3.685 | 1.312 |
|  | **ERα-HeLa-9903** | 1.783 | 3.065 | 2.038 | - | 19.459 | 2.798 |
|  | **MELN** | 2.775 | 2.806 | 2.857 | - | 9.048 | 0.248 |
|  | **LYES** | 2.085 | 3.255 | 0.830 | - | 0.757 | 1.306 |
|  | **LiBERA** | N.D. | N.D. | N.D. | N.D. | N.D. | N.D. |

TABLE S6. Limit of quantification (LOQ) of E1, E2, EE2, E3, NP and BPA using pYES and analysis of the water samples (WS1, WS2).

| Compound | LOQ (pg)* | LOQ (ng/L)  25 μL application volume  (1,000-fold enrichment) | WS1  (ng/L) | WS2  (ng/L) |
| --- | --- | --- | --- | --- |
| E1 | 2 | 0.080 | 2.3 | 4.5 |
| E2 | 0.5 | 0.020 | 0.25 | 0.5 |
| EE2 | 0.5 | 0.020 |  | 0.06 |
| E3 | 20 | 0.800 |  |  |
| NP | 120,000 | 4,800 |  |  |
| BPA | 5,000 | 200 |  |  |

*(Schoenborn et al., 2017)

TABLE S7. Quantification of E1, E2 and EE2 in extracted HM. 25 μL of the enriched sample (1000 x EQS) were analyzed by pYES. The average total amount for each compound is given in pg (right part, *n*=3, error = standard deviation). The respective concentrations of the compounds given by the measured amount divided by the application volume is expressed as x-fold EQS in the left part of the table.

| Sample  HM 1000 xEQS | nominal  x-fold EQS | measured  x-fold EQS | error  x-fold EQS | nominal amount  in 25 μL (pg) | measured amount  in 25 μL (pg) | error amount  in 25 μL (pg) |
| --- | --- | --- | --- | --- | --- | --- |
| E1 | 1,000 | 1,120 | 270 | 10 | 10.7 | 2.6 |
| E2 | 1,000 | 1,176 | 200 | 10 | 11.8 | 2.0 |
| EE2 | 1,000 | 1,120 | 270 | 0.875 | 0.98 | 0.24 |

TABLE S8. Quantification of E1, E2 and EE2 in extracted WS. The average total amount for each compound is given in pg/L and the relative maximal error in % (*n*=2).

| Substance | Concentration in WS1 (pg/L) | Rel. max error (n=2) (%) | Concentration in WS2 (pg/L) | Rel. max error (n=2) (%) |
| --- | --- | --- | --- | --- |
| E1 | 2,300 | 30% | 4,500 | 44 |
| E2 | 250 | 18% | 500 | 27 |
| EE2 | - | - | 60 | 16 |

TABLE S9. Identification of substances present in the water samples (W1, WS2) after pYES. Retention factors (RF), potential compounds and contribution to the total estrogenic effect (%) are indicated in the table.

| WS1 | | | WS2 | | |
| --- | --- | --- | --- | --- | --- |
| Retention factor (Rf) | Potential compound | Effect contribution (% to total estrogenicity) | Retention factor (Rf) | Potential compound | Effect contribution (% to total estrogenicity) |
| 0.18 | E3 | 5 | 0.17 | E3 | 5 |
| 0.27 | unknown | 7 | 0.19 | unknown | 8 |
| 0.41 | E2 | 40 | 0.28 | unknown | 14 |
| 0.66 | E1 | 48 | 0.41 | E2 | 31 |
|  | | | 0.55 | EE2 | 3 |
|  |  |  | 0.66 | E1 | 38 |

**Supplementary figures**

**
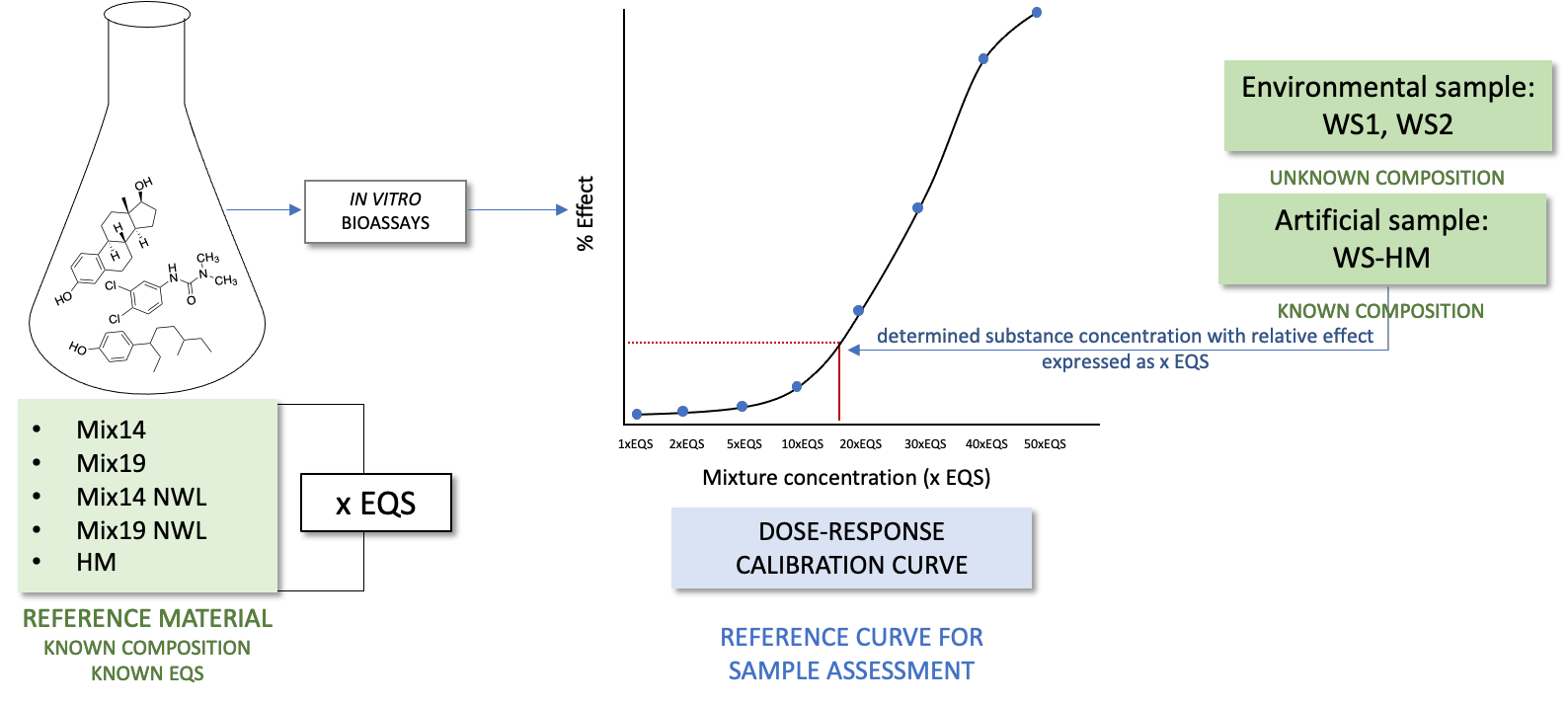
**

FIGURE S1. Schematic representation of water sample assessment (artificial or environmental) using reference materials.

FIGURE S2. Concentration-response curves generated with the RM (Mix14, Mix19, Mix14NWL, Mix19NWL and HM) and water samples (WS1 and WS2) using ER-GeneBLAzer. Estrogenic response (green) and cytotoxicity (black) in separated (right, middle) and combined logarithmic (left) plots showing 10% response relative to reference ER agonist and cell viability relative to control condition, respectively, are reported as EQS-fold or REF concentrations. Mix14, Mix19, Mix14NWL and Mix19NWL were prepared by mixing organic compounds in methanol and inorganic chemicals in 2% nitric acid. To prepare 1,000 x EQS HM, 3.5 mL of HM was added to 1L water and then enriched through SPE. Cytotoxicity of the WS was observed at concentrations higher than the estrogenicity EC_10_ values (WS1, EC10=48.5 REF; WS2, EC10=23.8 REF)

FIGURE S3. Effect-concentration of the water samples at 1 REF, WS1 (A), WS2 (B), WS-HM (C), extrapolated to the different reference mixtures. The concentration-response curves prepared for the water samples were used to determine the % of effect corresponding to a concentration 1 REF (not diluted or concentrated) in each bioassay. These values where then used to extrapolate the corresponding effect-concentration in the concentration-response curves prepared with the reference materials.


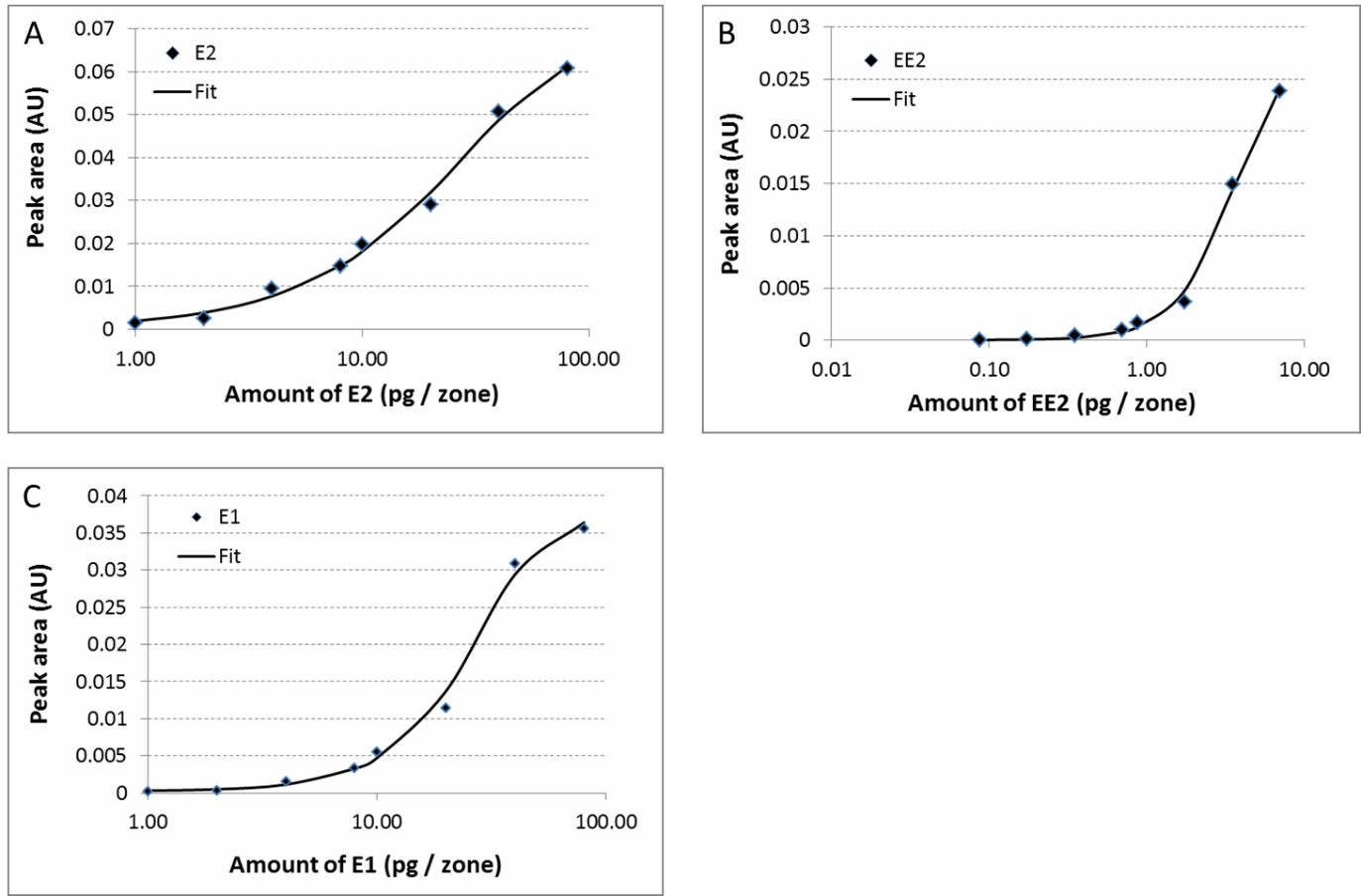


FIGURE S4. Dose-response relationship of **(A)** estradiol (E2), **(B)** ethinylestradiol (EE2) and **(C)** estrone (E1) in pYES. Average peak areas are plotted against the total mass applied per zone. The fitted function is indicated by the straight line.


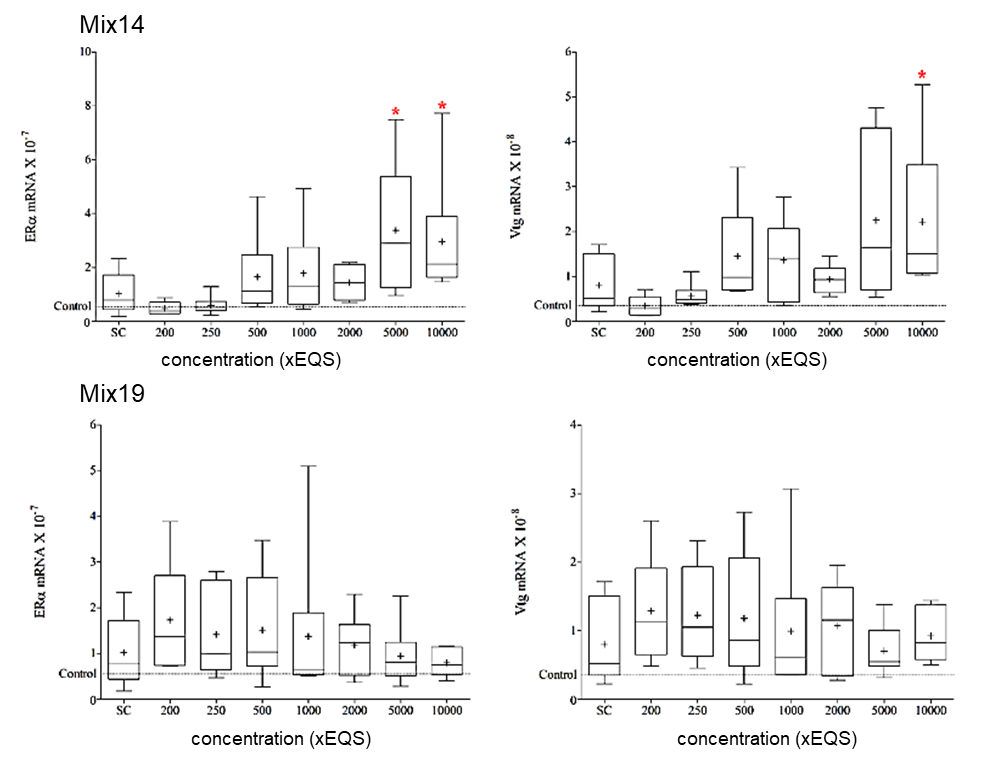


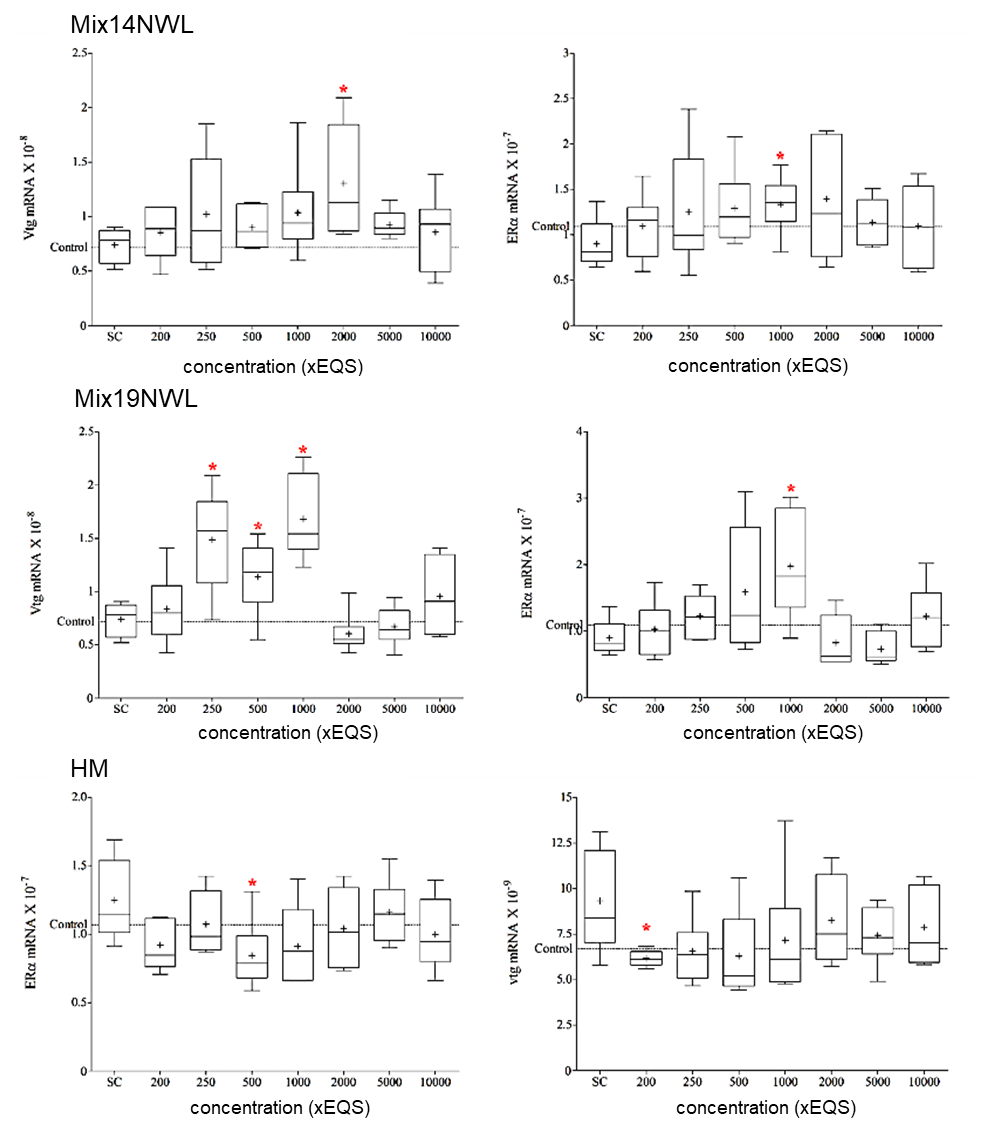


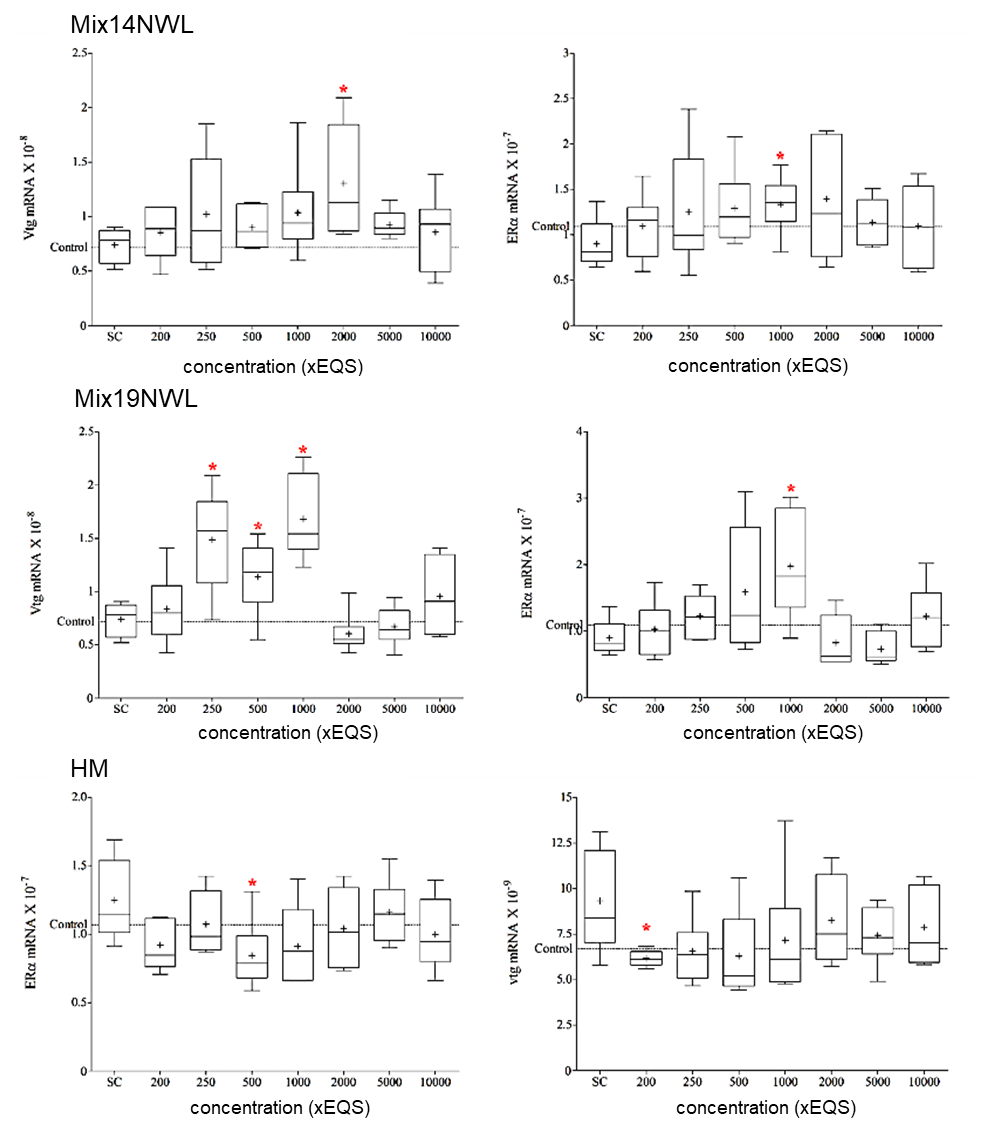


FIGURE S5. Gene expression analysis with quantitative real-time PCR of the molecular biomarkers erα and vtg after exposure of *Poeciliopsis lucida* hepatocellular carcinoma-derived PLHC-1 cell line to Mix14, Mix19, Mix14NP, Mix19NP and hormone mixture (HM). Results expressed as mRNA copies. Significantly different responses respect to the control are indicated by asterisks.

**Supplementary Materials and Methods**

***Composition of Reference Mixtures***

Five chemical mixtures were studied (i.e. Mix14, Mix19, Mix14NWL, Mix19NWL, and HM). Mix14 was composed of the priority substances atrazine, diuron, isoproturon and simazine (photosystem II inhibiting herbicides), benzo[a]pyrene (BaP) and fluoranthene (polycyclic aromatic hydrocarbons), cadmium and nickel (metals), DEHP (plasticizer), 4-nonylphenol (surfactant), chlorfenvinphos and chlorpyrifos (insecticides), 17β-estradiol (naturally occurring estrogen) and diclofenac (pain killer) which have been included in the first Watch List under the European Water Framework Directive (WFD) (Carvalho et al., 2014). In addition to the compounds listed above, Mix19 (Table S11) contained five emerging pollutants: bisphenol A (BPA, used for the production of plastics and food packaging products), carbamazepine (prescription medicine), sulfamethoxazole (a sulfonamide bacteriostatic antibiotic), triclosan (an antibacterial and antifungal agent) and DEET (an insect repellent), all at concentrations corresponding to their EQS value under river basin management plans. Variations of Mix14 and Mix19 without the Watch List (substances) E2 and diclofenac were also tested (Mix14NWL and Mix19NWL, respectively) (Tables S12 and S13). A hormone mixture (HM) containing the Watch List (WL) hormones (Table S15) at two different concentrations (i.e. 10,000 x EQS and 1,000 x EQS) was included in the study. For the preparation of the reference mixtures, a total of seven reference materials were produced (i.e., four organic, one inorganic and two hormone reference materials) as detailed in Tables S10-S14. The organic and hormone reference materials were prepared in methanol, while the inorganic reference material was prepared in water containing 2% nitric acid.

TABLE S10. Composition of the reference material for the preparation of Mix14.

| **Mix14** | |
| --- | --- |
| **RM076 (Organic Mixture 10 000 x EQS, methanol)** | **RM078 (Inorganic Mixture 10 000 x EQS, water 2% nitric acid)** |
| atrazine  benzo(a)pyrene  chlorfenvinphos  chlorpyrifos  DEHP  diclofenac  diuron  17-beta-estradiol  fluoranthene  isoproturon  4-nonylphenol  simazine | cadmium  nickel |

TABLE S11. Composition of the reference material for the preparation of Mix19.

| **Mix19** | |
| --- | --- |
| **RM077 (Organic Mixture 10 000 x EQS, methanol)** | **RM078 (Inorganic Mixture 10 000 x EQS, water 2% nitric acid)** |
| atrazine  benzo(a)pyrene  chlorfenvinphos  chlorpyrifos  DEHP  diclofenac  diuron  17-beta-estradiol  fluoranthene  isoproturon  4-nonylphenol  simazine  carbamazepine  sulfamethoxazole  triclosan  N,N-diethyl-m-toluamide  bisphenol A | cadmium  nickel |

TABLE S12. Composition of the reference material for the preparation of Mix14NWL.

| **Mix14NWL** | |
| --- | --- |
| **RM081 (Organic Mixture 10 000 x EQS, methanol)** | **RM078 (Inorganic Mixture 10 000 x EQS, water 2% nitric acid)** |
| atrazine  benzo(a)pyrene  chlorfenvinphos  chlorpyrifos  DEHP  diuron  fluoranthene  isoproturon  4-nonylphenol  simazine | cadmium  nickel |

TABLE S13. Composition of the reference material for the preparation of Mix19NWL.

| **Mix19NWL** | |
| --- | --- |
| **(Organic Mixture 10 000 x EQS, methanol)** | **RM078 (Inorganic Mixture 10 000 x EQS, water 2% nitric acid)** |
| atrazine  benzo(a)pyrene  chlorfenvinphos  chlorpyrifos  DEHP  diuron  fluoranthene  isoproturon  4-nonylphenol  simazine  carbamazepine  sulfamethoxazole  triclosan  N,N-diethyl-m-toluamide  bisphenol A | cadmium  nickel |

TABLE S14. Composition of the reference material for the preparation of the HM.

| **HM** | **HM WS** |
| --- | --- |
| **RM083 (Hormone Mixture 10 000 x EQS)** | **RM084 (Hormone Mixture 1000 x EQS)** |
| estrone (E1)  17beta-estradiol (E2)  17alpha-ethinylestradiol (EE2) | estrone (E1)  17beta-estradiol (E2)  17alpha-ethinylestradiol (EE2)  To be reconstituted in 1L water to create an artificial water sample of 1 x EQS (HM WS) |

***Stability and homogeneity studies***

The seven reference materials were produced by ISPRA (i.e. ISPRA RM076, ISPRA RM077, ISPRA RM078, ISPRA RM081, ISPRA RM082, ISPRA RM083, ISPRA RM084) according to ISO 17034 (ISO 17034:2016). Both homogeneity and stability were assessed according to ISO Guide 35 (2017) on ISPRA RM076, ISPRA RM077, ISPRA RM081, ISPRA RM082, ISPRA RM083 and ISPRA RM084. In particular, a short-term stability study was carried out to simulate degradation under storage and transport conditions during the EU-wide study exercise. The reference materials have been dispatched in winter time. Although under dry ice, a temperature of 24°C ± 4°C was chosen to take into account any improper shipment and -20°C ± 4°C ± 4°C and 4°C ± 2°C as reference temperatures for the organic and inorganic mixtures respectively. A temperature of 24°C ± 4°C was chosen to take into account any improper shipment and -20°C ± 4°C as reference temperature.

For stability assessment, an experimental design according to the isochronous study was planned. This approach minimizes the variations in analytical response as a function of time (Bonas et al., 2003; ISO17034, 2016; ISOGuide35, 2017; Lamberty et al., 1998; Linsinger et al., 2001). The present study was carried out considering the time points of one month, two months and three months (in the period 01/12/2018-01/03/2019). Five units for each time point were analyzed in triplicate and the results were plotted against time. A screening of any suspicious data was performed and the regression line for the property versus time was calculated. Both slope and regression line were tested for statistically significant trends.

For homogeneity study of reference materials, the results obtained within the isochronous measurements in the absence of a trend over time were assessed by applying a two-way ANOVA approach (a one-way ANOVA approach was followed for unbalanced samples size in case of detection of suspicious data).

*Organic mixture for Mix14 (RM076)*

All relevant substances were determined by means of a UHPLC/MS-MS and the obtained data were plotted in function of time (Figure S6). Benzo(a)pyrene, DHEP, diclofenac, fluoranthene, 4-nonylphenol and 17-β-estradiol (E2) were not determined in this reference material. The stability and homogeneity assessment were based on the measurements carried out in previous reference materials productions similar to the present organic mixture in terms of composition and concentration (Carvalho et al., 2014). Overall, the properties of interest are stable and homogenous in the present reference material.


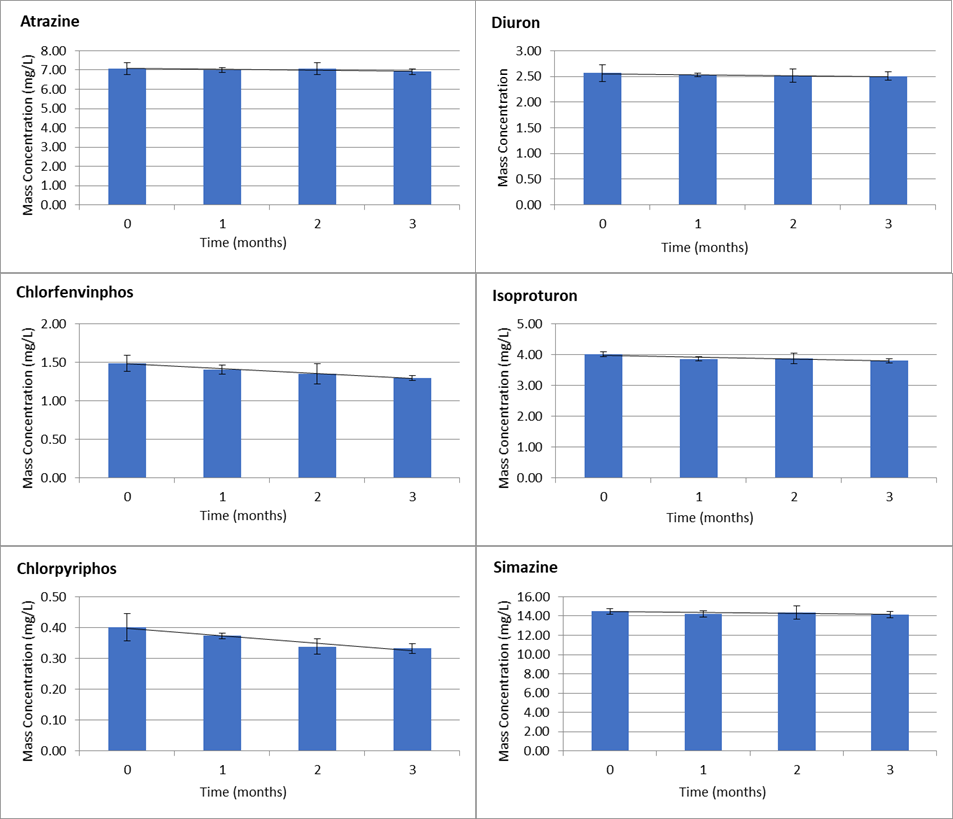


FIGURE S6. Stability of compounds determined in the reference organic mixture for Mix14. Plots are shown for substances assessed through UHPLC/MS-MS in this study.

Upon the stability assessment in the reference organic mixture, the test for significance failed in the case of atrazine, chlorfenvinphos, chlorpyrifos and diuron as confirmed by data trends. However, the degree of instability was still acceptable considering percentage of repeatability and low standard deviation (Table S15). Regarding the homogeneity assessment, the obtained results showed that all the compounds were homogenous in the tested material, therefore the reference organic mixture was considered fit for purpose.

TABLE S15. Concentrations of substances composing the reference organic mixture for Mix14. The values are based on the present and previous studies and listed with the corresponding associated expanded uncertainties (U), assuming k=2 as coverage factor. Repeatability and standard deviation (SD) are reported for substances that lacked statistical significance in the stability assessment.

| **Compound** | **Concentration**  **(mg/L)** | **U, k=2**  **(mg/L)** | **Repeatability**  **(%)** | **SD**  **(%)** |
| --- | --- | --- | --- | --- |
| Atrazine | 6.00 | 0.40 | 6.0 | 2.0 |
| BaP | 0.00177 | 0.00021 |  |  |
| Chlorfenvinphos | 1.008 | 0.094 | 8.0 | 1.0 |
| Chlorpyriphos | 0.304 | 0.031 | 7.0 | 4.0 |
| DHEP | 12.7 | 3.2 |  |  |
| Diclofenac | 0.97 | 0.16 |  |  |
| Diuron | 2.13 | 0.14 | 5.0 | 3.0 |
| Fluoranthene | 0.0642 | 0.0054 |  |  |
| Isoproturon | 3.01 | 0.19 |  |  |
| Nonylphenol | 3.01 | 0.50 |  |  |
| Simazine | 10.00 | 0.68 |  |  |
| β-Estradiol | 0.00390 | 0.00061 |  |  |

*Organic mixture for Mix19 (RM077)*

All substances in the present organic mixture were determined by UHPLC/MS-MS at ISPRA with the exception of 17-β-estradiol (E2) the concentration of which was determined by ARPA Lombardia (Figure S7). Benzo(a)pyrene, DHEP, diclofenac, fluoranthene and 4-nonylphenol, bisphenol A, carbamazepine, DEET, sulfamethoxazole and triclosan were not determined in this reference material as their stability and homogeneity assessment was performed based on the measurements carried out in previous reference materials productions similar in terms of composition and concentration. Overall, the analytes were stable and homogenous in this reference material (Table S16).


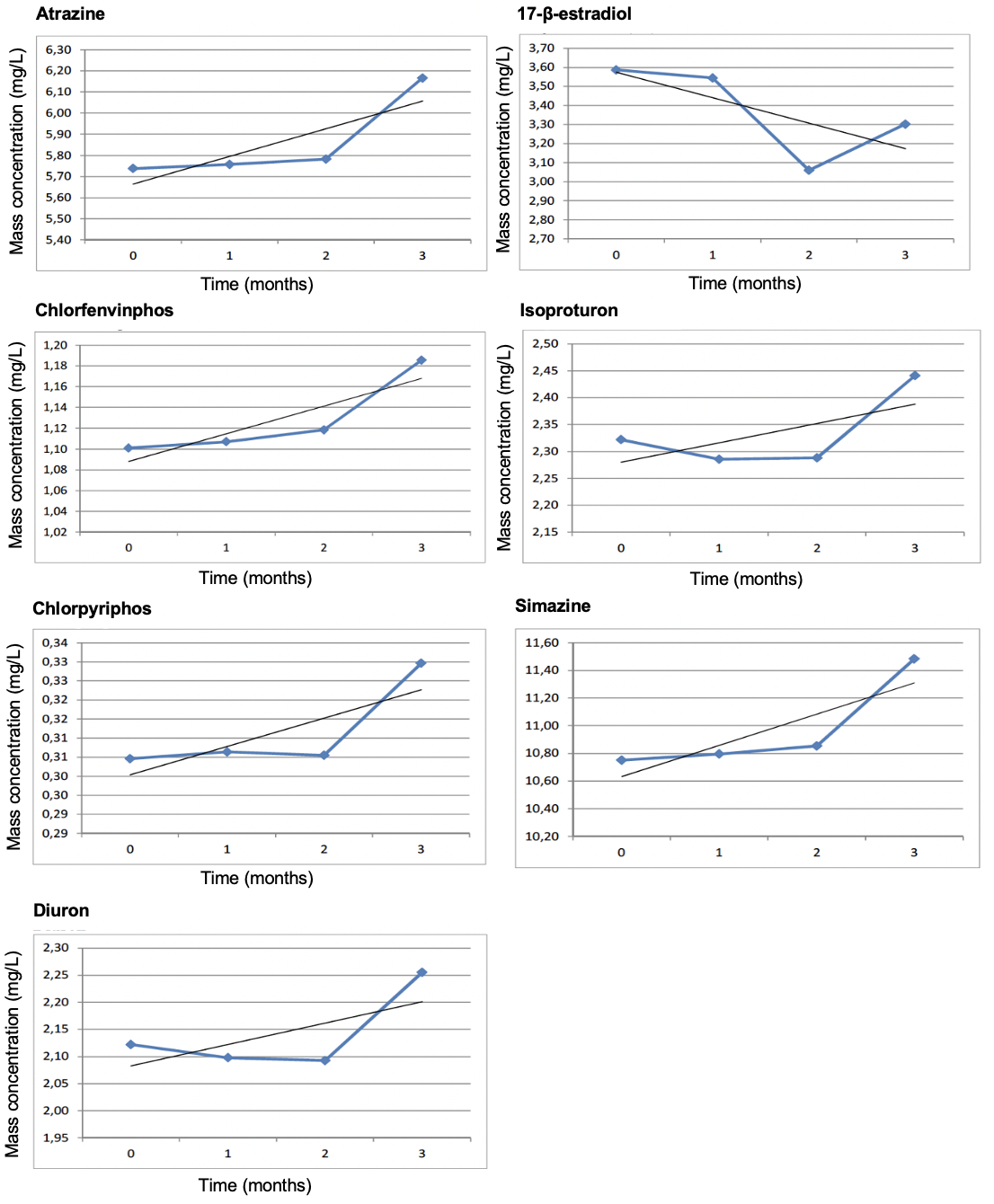


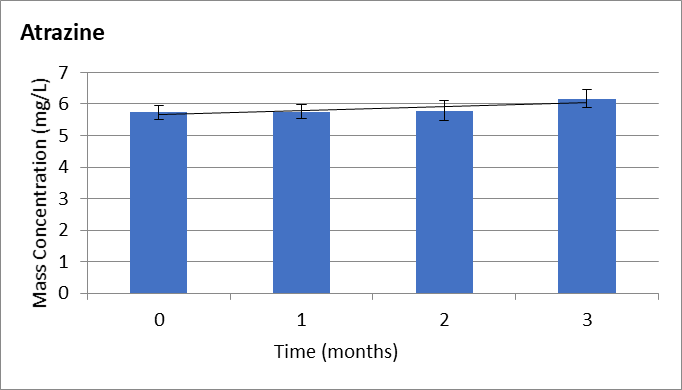

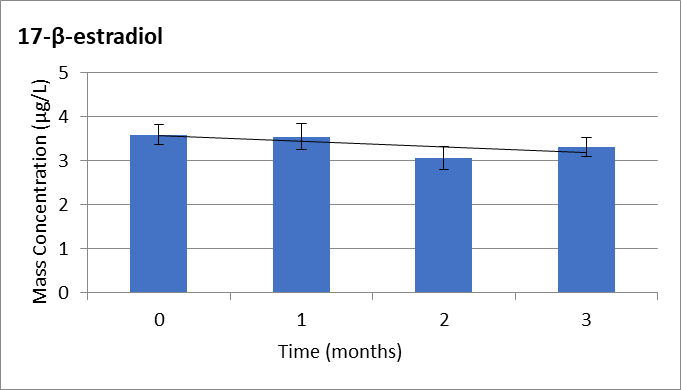

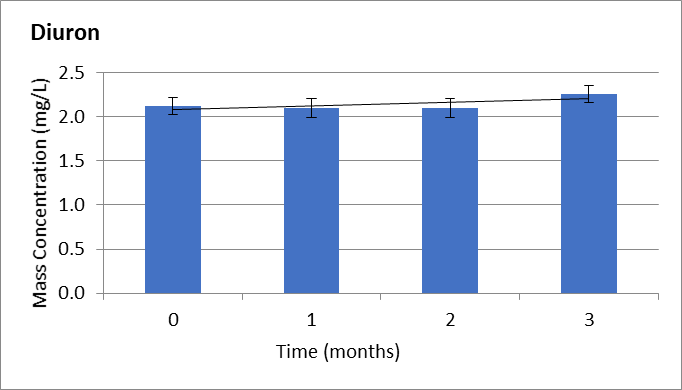

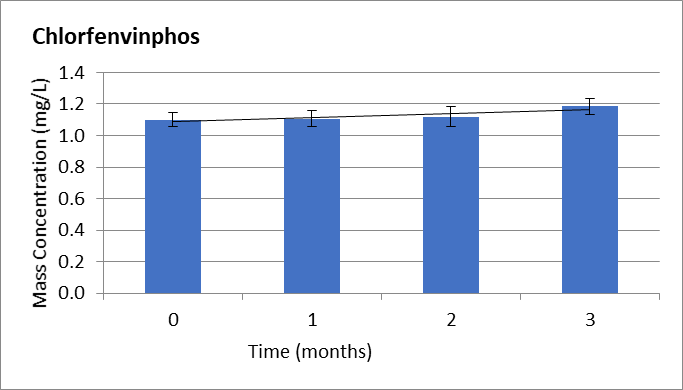

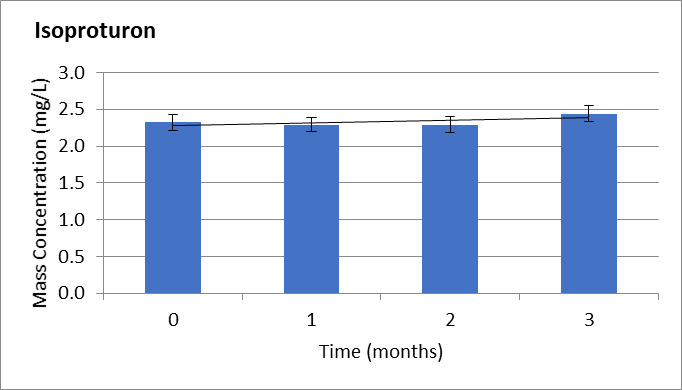

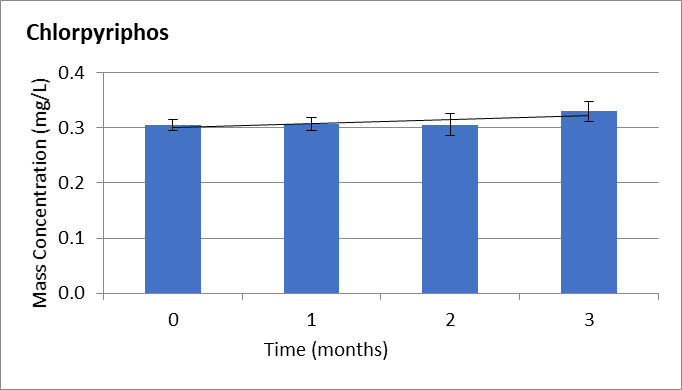

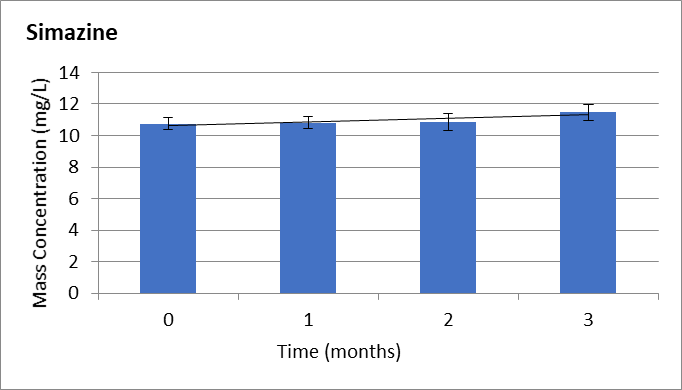


FIGURE S7. Stability of compounds determined in the reference organic mixture for Mix19. Plots of mass concentration in function of time are shown for substances assessed in this study using UHPLC/MS-MS.

TABLE S16. Concentrations of substances composing the reference organic mixture for Mix19. The values are based on the present and previous studies and listed with the corresponding associated expanded uncertainties (U), assuming k=2 as coverage factor. Substances absent in the organic mixture for Mix14 are reported under the double line.

| **Compound** | **Concentration**  **(mg/L)** | **U, k=2**  **(mg/L)** |
| --- | --- | --- |
| Atrazine | 6.00 | 0.63 |
| BaP | 0.00177 | 0.00021 |
| Chlorfenvinphos | 1.01 | 0.11 |
| Chlorpyriphos | 0.304 | 0.035 |
| DHEP | 12.7 | 3.2 |
| Diclofenac | 0.99 | 0.17 |
| Diuron | 2.13 | 0.25 |
| Fluoranthene | 0.0642 | 0.0054 |
| Isoproturon | 3.01 | 0.34 |
| Nonylphenol | 3.01 | 0.50 |
| Simazine | 10.00 | 0.99 |
| β-Estradiol | 0.00390 | 0.00079 |
| Bisphenol A | 15.5 | 1.8 |
| Carbamazepine | 5.0 | 1.0 |
| DEET | 454 | 100 |
| Sulfamethoxazole | 6.0 | 1.3 |
| Triclosan | 0.196 | 0.033 |

*Inorganic mixture (RM078)*

Ni and Cd were not determined in this reference material. The stability and homogeneity assessment of these analytes are based on the measurements carried out in previous similar reference materials production and on literature studies (Table S17).

TABLE S17. Concentrations of metals composing the reference inorganic mixture for Mix14 and Mix19. The values are based on previous studies and listed with the corresponding associated expanded uncertainties (U), assuming k=2 as coverage factor.

| **Compound** | **Concentration**  **(mg/L)** | **U, k=2**  **(mg/L)** |
| --- | --- | --- |
| Ni | 40.0 | 3.2 |
| Cd | 0.80 | 0.06 |

*Organic mixture without pharmaceuticals for Mix14 NWL (RM081)*

Assessment of stability and homogeneity was performed for atrazine, chlorfenvinphos, chlorpyriphos, diuron, isoproturon and simazine (Figure S8). Values relative to benzo(a)pyrene, DHEP, fluoranthene and 4-nonylphenol are based on the measurements carried out in previous reference materials productions similar to the present mixture in terms of composition and concentration (Table S18). All analytes resulted stable and homogenous in this reference material.


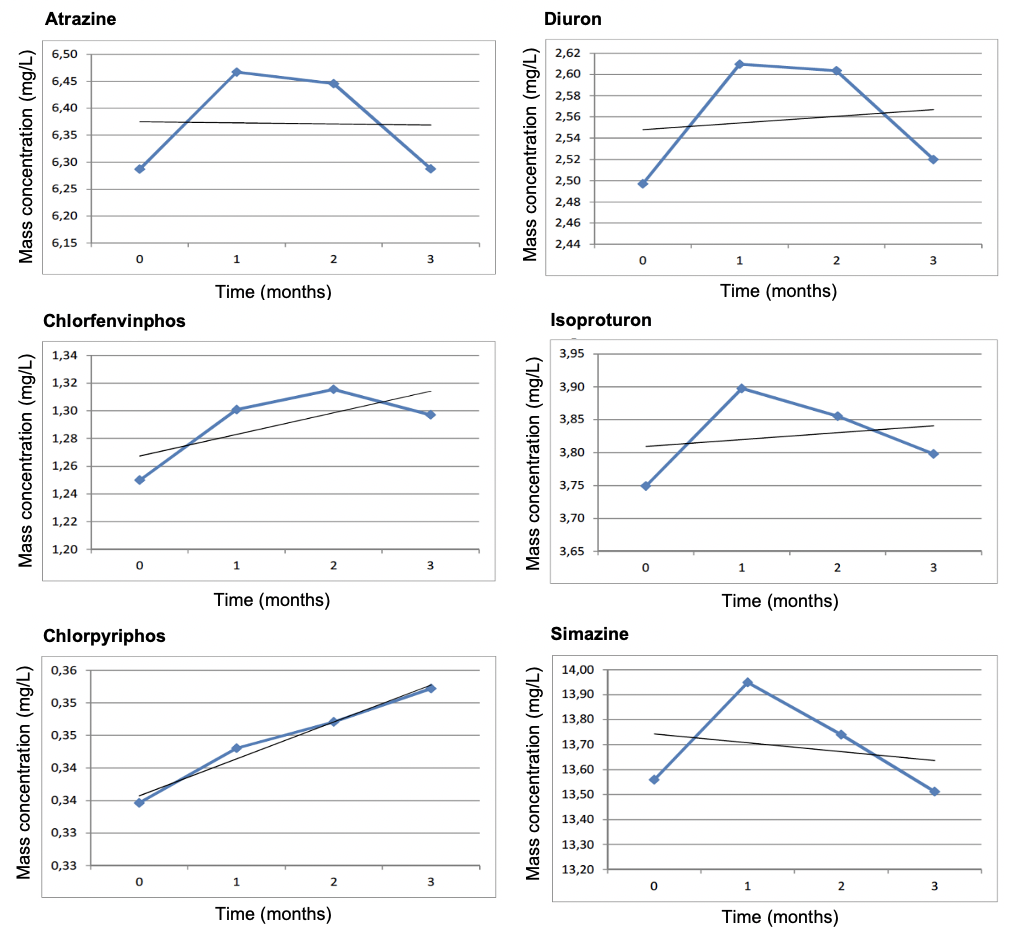


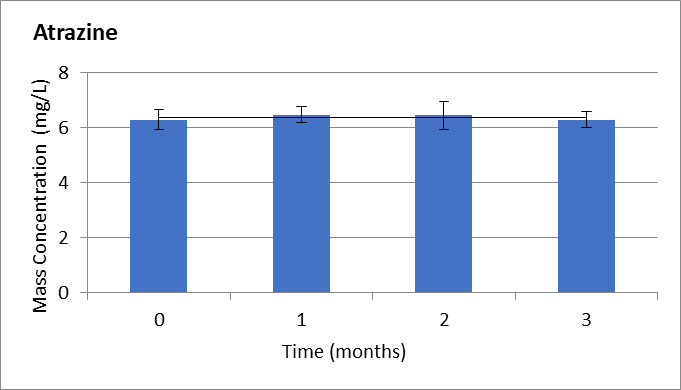

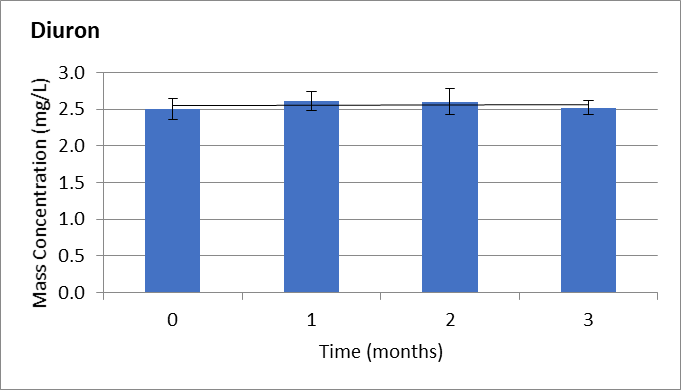

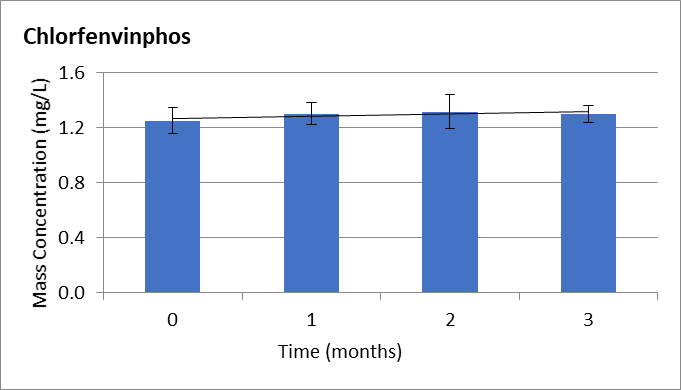

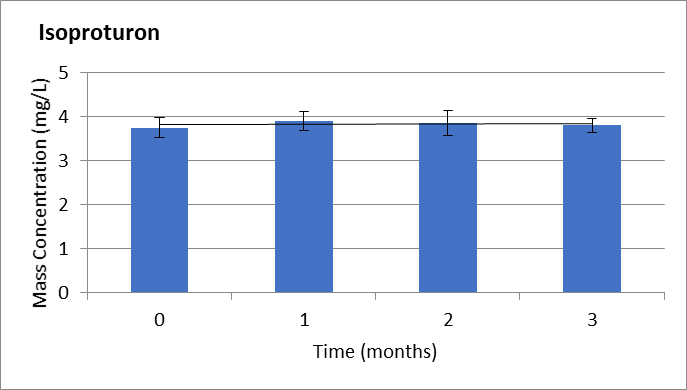

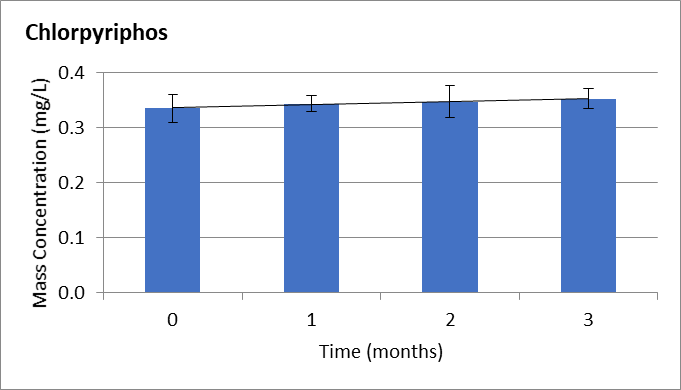

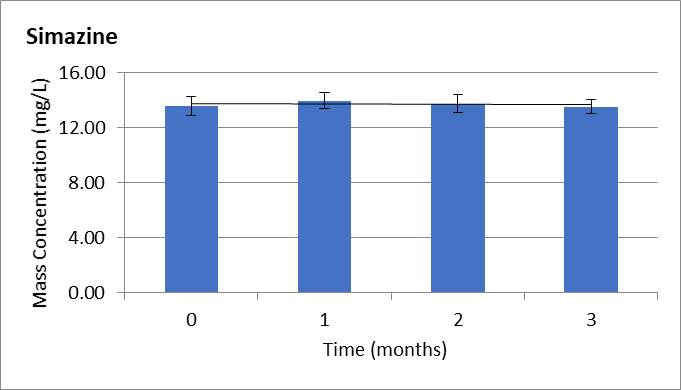


FIGURE S8. Stability of compounds determined in the reference organic mixture for Mix14 NWL. Plots of mass concentration in function of time are shown for substances assessed in this study by UHPLC/MS-MS.

TABLE S18. Concentrations of substances composing the reference organic mixture for Mix14 NWL. The values are based on the present and previous studies and listed with the corresponding associated expanded uncertainties (U), assuming k=2 as coverage factor.

| **Compound** | **Concentration**  **(mg/L)** | **U, k=2**  **(mg/L)** |
| --- | --- | --- |
| Atrazine | 6.08 | 0.79 |
| BaP | 0.00177 | 0.00021 |
| Chlorfenvinphos | 1.01 | 0.10 |
| Chlorpyriphos | 0.304 | 0.024 |
| DHEP | 12.7 | 3.2 |
| Diuron | 2.13 | 0.20 |
| Fluoranthene | 0.0642 | 0.0054 |
| Isoproturon | 3.01 | 0.22 |
| Nonylphenol | 3.01 | 0.50 |
| Simazine | 9.60 | 0.80 |

*Organic mixture without pharmaceuticals for Mix19 NWL (RM082)*

Stability and homogeneity of compounds were determined as shown in Figure S9. For benzo(a)pyrene, DHEP, fluoranthene and 4-nonylphenol, bisphenol A, carbamazepine, DEET, sulfamethoxazole and triclosan, relative values are based on the measurements carried out in previous reference materials productions similar in terms of composition and concentration (Table S19).


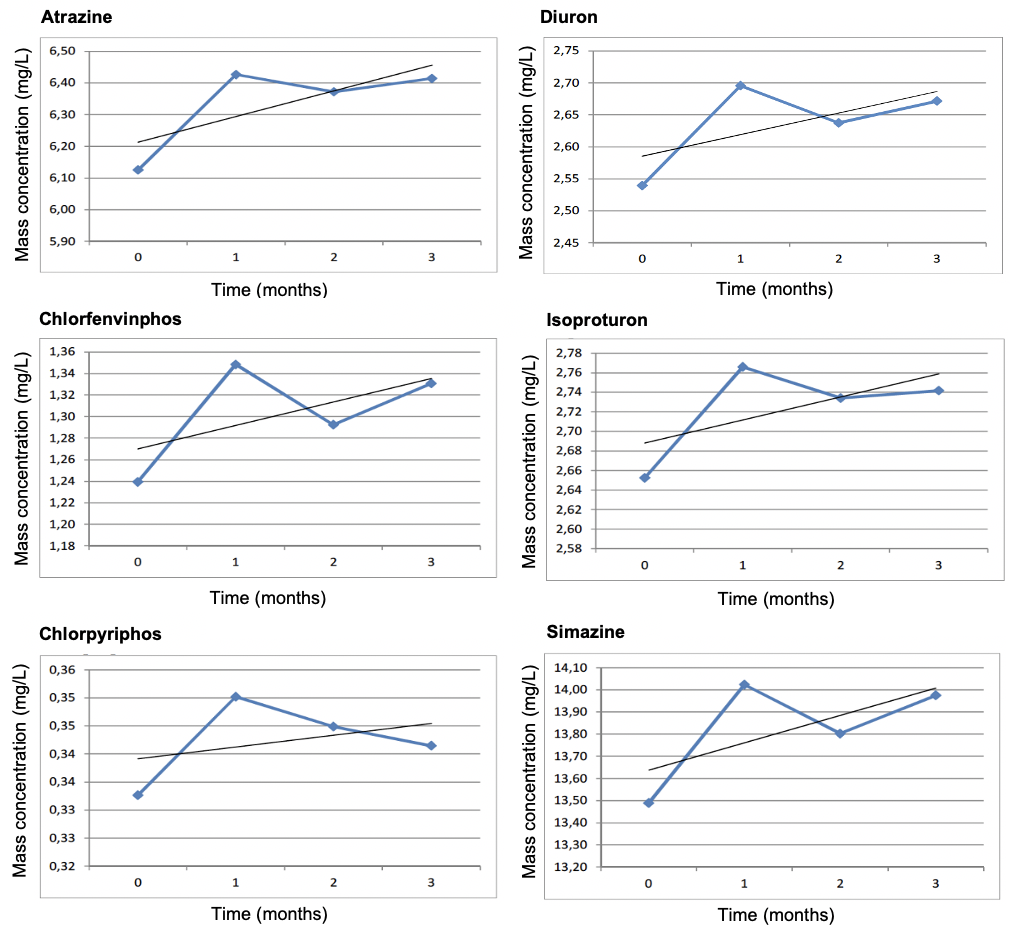
+


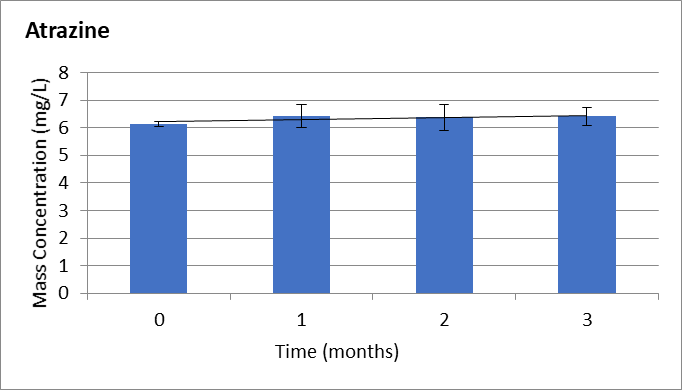

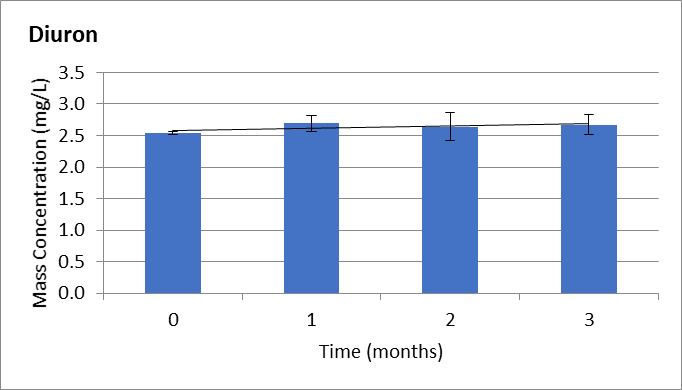

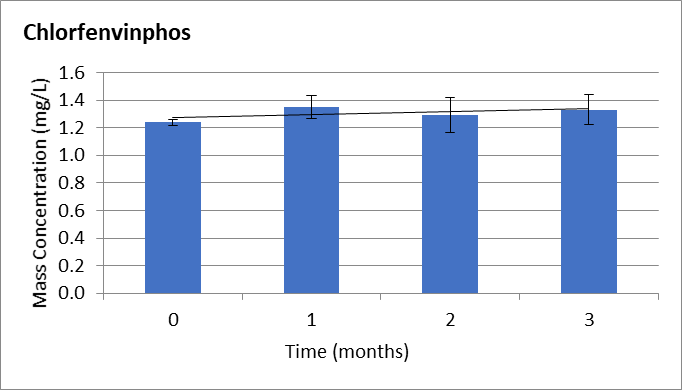

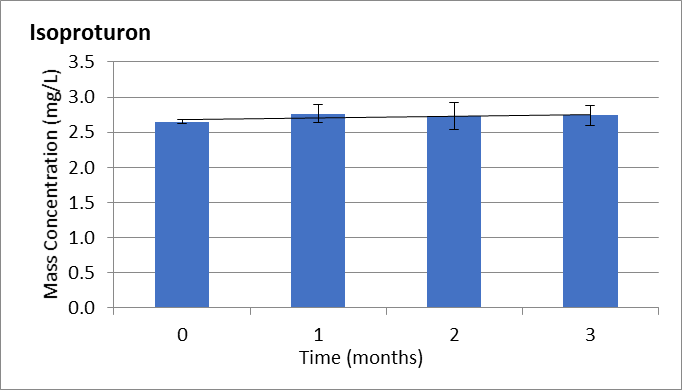

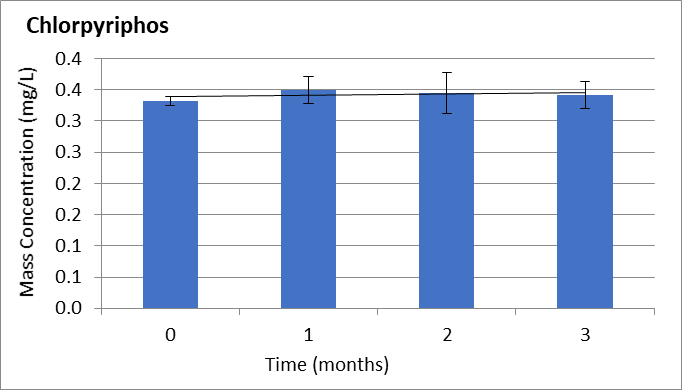

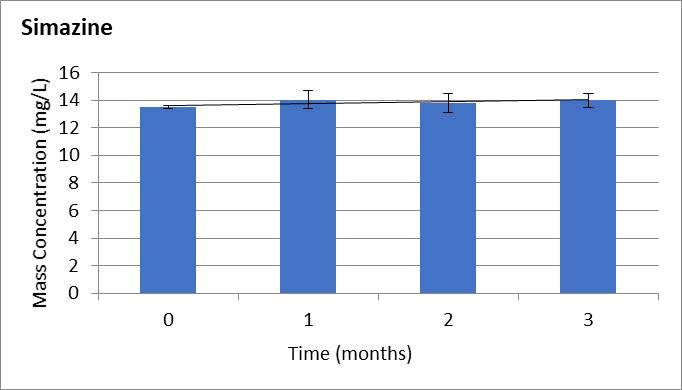


FIGURE S9 Stability of compounds determined in the reference organic mixture for Mix19 NWL. Plots of mass concentration in function of time are shown for substances assessed in this study by UHPLC/MS-MS.

TABLE S19. Concentrations of substances composing the reference organic mixture for Mix19 NWL. The values are based on the present and previous studies and listed with the corresponding associated expanded uncertainties (U), assuming k=2 as coverage factor. Substances absent in the organic mixture for Mix14 NWL are reported under the double line.

| **Compound** | **Concentration**  **(mg/L)** | **U, k=2**  **(mg/L)** |
| --- | --- | --- |
| Atrazine | 6.08 | 0.84 |
| BaP | 0.00177 | 0.00021 |
| Chlorfenvinphos | 1.01 | 0.15 |
| Chlorpyriphos | 0.304 | 0.037 |
| DHEP | 12.7 | 3.2 |
| Diuron | 2.13 | 0.22 |
| Fluoranthene | 0.0642 | 0.0054 |
| Isoproturon | 3.01 | 0.24 |
| Nonylphenol | 3.01 | 0.50 |
| Simazine | 9.60 | 0.80 |
| Bisphenol A | 3.10 | 0.37 |
| Carbamazepine | 5.0 | 1.0 |
| DEET | 454 | 100 |
| Sulfamethoxazole | 6.0 | 1.3 |
| Triclosan | 0.196 | 0.033 |

*Hormone mixture x10,000 (RM083)*

17-β-estradiol (E2), estrone (E1) and 17-α-ethinylestradiol (EE2) were determined by ARPA Lombardia through UHPLC/MS-MS. The analytes resulted stable and homogenous in the present reference material (Figure S10 and Table S20).


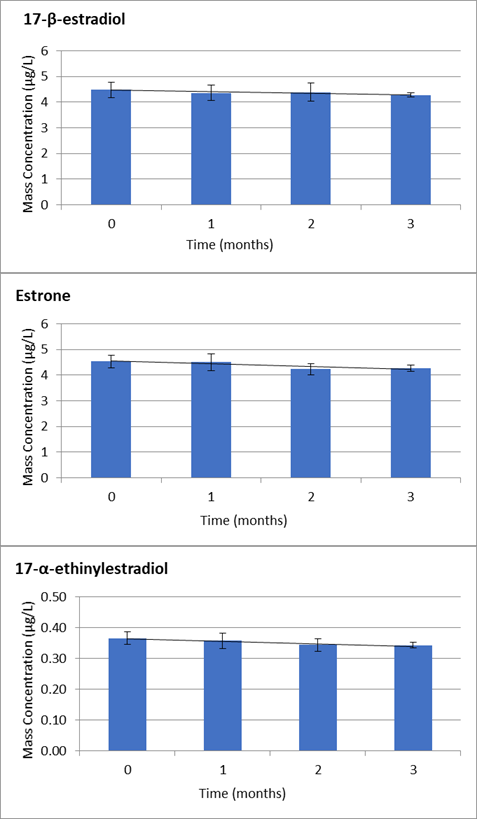


FIGURE S10. Stability of hormones determined in the reference hormone mixture x10000.

TABLE S20. Concentrations of hormones composing the reference hormone mixture x10,000. The values are indicated with the corresponding associated expanded uncertainties (U), assuming k=2 as coverage factor.

| **Compound** | **Concentration**  **(mg/L)** | **U, k=2**  **(mg/L)** |
| --- | --- | --- |
| E2 | 0.00390 | 0.00044 |
| E1 | 0.0040 | 0.0004 |
| EE2 | 0.00032 | 0.00004 |

*Hormone mixture x1,000 (RM084)*

The present mixture was obtained by diluting the bulk of hormone mixture x10,000 by a factor of 10. Relative concentrations of E1, E2 and EE2 were determined by ARPA Lombardia by using UHPLC/MS-MS showing the stability of relevant properties in this reference material (Figure S11 and Table S21). The considered data sets failed the ANOVA test within the homogeneity assessment which was probably due to the low mass concentrations of the analytes close to the LOQs of the applied measurement method. At this level of concentration, the corresponding measurement uncertainties, though still acceptable, are high. Considering that the hormone mixture x1,000 is a dilution of the hormone mixture x10,000 resulting itself stable and homogenous, the reference material is considered fit for purpose.


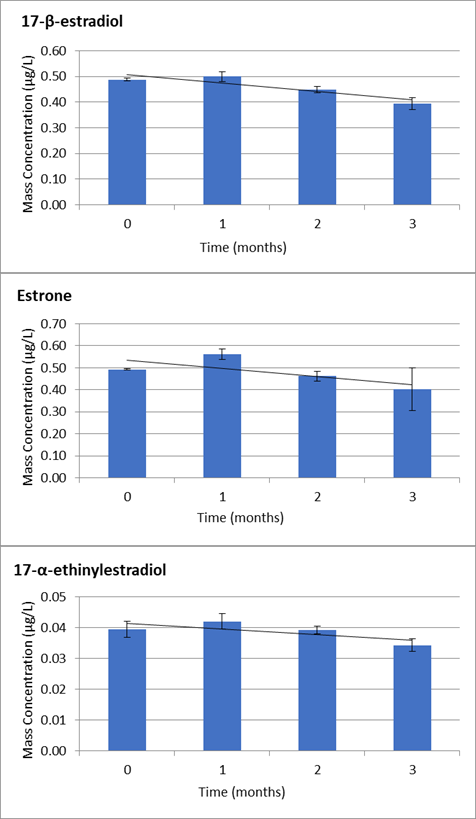


FIGURE S11. Stability of hormones determined in the reference hormone mixture x1,000.

TABLE S21. Concentrations of hormones composing the reference hormone mixture x1,000. The values are indicated with the corresponding associated expanded uncertainties (U), assuming k=2 as coverage factor.

| **Compound** | **Concentration**  **(mg/L)** | **U, k=2**  **(mg/L)** |
| --- | --- | --- |
| E2 | 0.00039 | 0.00010 |
| E1 | 0.00040 | 0.00016 |
| EE2 | 0.000032 | 0.000008 |

***Analytical methods for water samples***

Benzo[a]pyrene (BaP), chlorpyrifos, DEHP, fluoranthene, 4-nonylphenol, triclosan and BPA were determined by LC-MS/MS electrospray ionization (ESI, neg/pos) direct analysis (Table S22) after liquid-liquid microextraction (1L of sample with 4 mL of toluene), evaporation to dryness, addition of toluene PCB 209 as internal standard to 100 μL of final volume.

TABLE S22. Concentration of substances in water samples analyzed by LC-MS/MS ESI following the protocol for liquid-liquid microextraction.

| **Compound** | **Instrumental LOD**  **(ng/L)** | **Calibration points in water**  **(ng/L)** |
| --- | --- | --- |
| Benzo(a)pirene | 0.05 | 0.2-0.4-10 |
| Chlorpyrifos | 9 | 10-20-50 |
| DEHP | 390 | 100-200-500 |
| Fluoranthene | 2 | 0.2-0.4-1 |
| 4-nonylphenol | 90 | 100-200-500 |
| Triclosan | 20 | 20-50-100 |
| Bisphenol A | 1.5 | 1.5-3-5 |

LOD: limit of detection

Atrazine, chlorfenvinphos, diclofenac, diuron, E2, isoproturon, simazine, carbamazepine, sulfamethoxazole and DEET were determined after addition of methanol (5 mL in 1L of sample) to water samples (Table S23), filtration on 0.45 μm membrane, addition of atrazine-D5 as internal standard for ESI positive mode and MCPA-D3 for ESI negative mode, and carbamazepine-C13 for carbamazepine determination.

The extraction of E2 was carried out according to internal method by the extraction of 1L sample with SPE-DEX (Horizon Technology, Italy) using a sandwich of HLB+C18 disks and elution with 20 mL of ethyl acetate-methanol-MTBE (1:1:1) mix. The solution was then evaporated to 500 μL and analyzed in ESI negative mode.

Finally, the metals Ni and Cd were analyzed by ICP-MS (Table S24).

TABLE S23. Concentration of substances in water samples analyzed by LC-MS/MS ESI following SPE-DEX extraction or membrane filtration protocols.

| **Compound** | **LOD (validated method) (µg/L)** | **Calibration points in water**  **(µg/L)** |
| --- | --- | --- |
| Atrazine | 0.05 | 0.05-0.1-0.25-0.5-1-2 |
| Chlorfenvinphos | 0.05 | 0.05-0.1-0.25-0.5-1-2 |
| Diclofenac | 0.05 | 0.05-0.1-0.25-0.5-1-2 |
| Diuron | 0.05 | 0.05-0.1-0.25-0.5-1-2 |
| 17-β-estradiol | 0.0004 | 0.00025-0.000.5-0.001-0.025-0.05 |
| Isoproturon | 0.05 | 0.05-0.1-0.25-0.5-1-2 |
| Simazine | 0.05 | 0.05-0.1-0.25-0.5-1-2 |
| Carbamazepine | 0.1 | 0.1-0.25-0.5-1-2 |
| Sulfamethoxazole | 0.1 | 0.1-0.25-0.5-1-2 |
| DEET | 0.05 | 0.05-0.1-0.25-0.5-1-2 |

LOD: limit of detection

TABLE S24. Concentration of metals in waters samples analyzed by ICP-MS.

| **Compound** | **LOD (validated method)**  **(µg/L)** | **Calibration points in water**  **(µg/L)** |
| --- | --- | --- |
| Ni | 1.0 | 0.025-0.05-0.1-0.2-0.5-1 |
| Cd | 0.05 | 0.025-0.05-0.1-0.2-0.5-1 |

LOD: limit of detection

***Cytotoxicity of the reference mixtures and water samples in the cell-based bioassays***

Cytotoxicity of the RM, HM and WS extract was assessed in parallel to the cell-based reporter gene bioassays according to the individual protocols of the test performers (e.g. estrogenic and anti-estrogenic effects in hERα-HeLa-9903 cells; androgenic, anti-androgenic and glucocorticoid effects in MDA-Kb2).. The cytotoxicity was determined by using resazurin assay according to Schirmer et al. 1998 (Schirmer et al., 1998) with slight modifications. Briefly, cells were cultured and exposed to solvent control and samples. After a 24 h incubation period, resazurin (Biotech, SERVA, Germany) was diluted with DMEM to prepare resazurin solution at a concentration of 0.1% v/v and added to wells (100 μL/well). Cells were incubated for 1 h at 37°C. The intensity of fluorescence (λ_ex_540/λ_em_590 nm) was measured using POLARstar OPTIMA microplate spectrophotometer (BMG Labtech, Germany). RM, HM and WS extract dilutions were considered cytotoxic if the absorbance at a tested concentration was less than three times the standard deviation of the absorbance of solvent control. The assays were conducted in 96-well microplates and included at least three dilutions in three replicates to characterize a dose-response curve for each material and WS. All reference materials and WS extracts were tested in at least two independent experiments. For ER-GeneBLZer assay cytotoxicity was quantified through change in confluency during the exposure quantified by an IncuCyte S3 live cell imaging system (Essen BioScience, Ann Arbor, Michigan, USA) as described by Escher et al. (2019).

**Supplementary references**

Bonas G, Zervou M, Papaeoannou T, Lees M, 2003. ”SoftCRM”: a new software for the Certification of Reference Materials. Accreditation and Quality Assurance; 8: 101-107.

Carvalho RN, Arukwe A, Ait-Aissa S, Bado-Nilles A, Balzamo S, Baun A, et al, 2014. Mixtures of Chemical Pollutants at European Legislation Safety Concentrations: How Safe Are They? Toxicological Sciences; 141: 218-233. https://doi.org/10.1093/toxsci/kfu118.

Escher BI, Glauch L, König M, Mayer P, Schlichting R, 2019. Baseline Toxicity and Volatility Cutoff in Reporter Gene Assays Used for High-Throughput Screening. Chemical Research in Toxicology; 32: 1646-1655. https://doi.org/10.1021/acs.chemrestox.9b00182.

ISO17034, 2016. General requirements for the competence of reference material producers.

ISOGuide35, 2017. Reference materials — Guidance for characterization and assessment of homogeneity and stability.

Lamberty A, Schimmel H, Pauwels J, 1998. The study of the stability of reference materials by isochronous measurements. Fresenius' Journal of Analytical Chemistry; 360: 359-361. https://doi.org/10.1007/s00216005071.

Linsinger TPJ, Pauwels J, van der Veen AMH, Schimmel H, Lamberty A, 2001. Homogeneity and stability of reference materials. Accreditation and Quality Assurance; 6: 20-25. https://doi.org/10.1007/s007690000261.

Schirmer K, Chan AGJ, Greenberg BM, Dixon DG, Bols NC, 1998. Ability of 16 priority PAHs to be photocytotoxic to a cell line from the rainbow trout gill. Toxicology; 127: 143-155. https://doi.org/10.1016/s0300-483x(98)00031-6.

Schoenborn A, Schmid P, Bräm S, Reifferscheid G, Ohlig M, Buchinger S, 2017. Unprecedented sensitivity of the planar yeast estrogen screen by using a spray-on technology. Journal of Chromatography A; 1530: 185-191. https://doi.org/10.1016/j.chroma.2017.11.009.
